# Methyl Radical Addition Reactions to C=X Double Bonds

**DOI:** 10.1021/acs.joc.5c01157

**Published:** 2025-07-21

**Authors:** Yuman Hordijk, Bart Waaijer, Christopher B. Kelly, Trevor A. Hamlin

**Affiliations:** † Department of Chemistry and Pharmaceutical Sciences, 1190 Amsterdam Institute for Molecular and Life Sciences (AIMMS), Vrije Universiteit Amsterdam De Boelelaan 1108, Amsterdam 1081 HZ, The Netherlands; ‡ Discovery Process Research, Johnson & Johnson Innovative Medicine, Spring House, Pennsylvania 19477, United States

## Abstract

Radical additions to olefins and related π-systems are essential tools in the modern synthetic toolbox for forging C–C and C–X bonds. We report a systematic DFT study (ZORA-(U)­OLYP/TZ2P) on methyl radical (H_3_C^•^) additions to H_2_C=X substrates, where X is varied across elements from the tetrel (group 14: C, Si, Ge), pnictogen (group 15: N, P, As), and chalcogen (group 16: O, S, Se) groups. Our analysis reveals clear periodic trends: addition barriers at carbon decrease, and those at X increase from tetrels to chalcogens; reaction energies become less favorable across a period but more favorable down a group. Regioselectivity favors addition at X except for X = CH_2_, NH, and O, where carbon attack dominates—consistent with experimental data. These trends arise from a balance of orbital interactions and Pauli repulsion, with the latter emerging as a key, yet underappreciated, factor governing radical reactivity and regioselectivity in π-systems.

## Graphical abstract

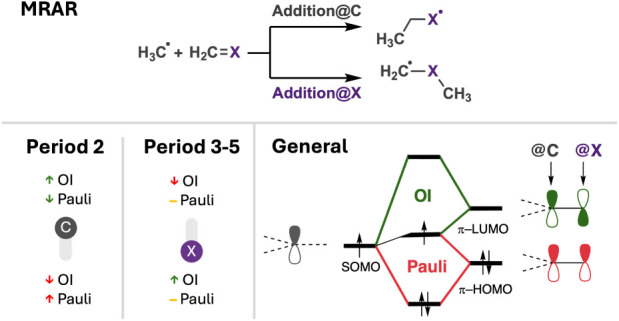

## Introduction

Radical addition reactions (RARs) represent a cornerstone of organic chemistry and play significant roles in various biological processes.^1^ These reactions involve the attack of a radical species on π-systems to form new radical adducts.^2^ These adducts can then be paired with secondary radical events (e.g., hydrogen atom transfer (HAT) or halogen atom transfer (XAT)) or as part of complex cycles (photocatalytic, reductive, etc.) to engage in polar reactivity. Despite the wealth of experimental studies exploring RARs,^3^ only a limited number of rigorous computational investigations exist to elucidate the fundamental driving forces governing the principles of reactivity and selectivity in the RAR manifold.^4^

In this study, we examine computationally the generic and ubiquitous methyl radical (H_3_C^•^) addition reactions to substrates of the general form H_2_C=X, where X encompasses a systematic set from Periods 2–5 and Groups 14–16 in the periodic table (CH_2_, SiH_2_, GeH_2_, SnH_2_, NH, PH, AsH, SbH, O, S, Se, Te, see Scheme 1). These reactions can proceed via two distinct pathways: methyl radical addition to the CH_2_ group (addition@C) or the X group (addition@X), yielding regioisomeric radical adducts. The objectives of this work are 2-fold: first, to rationalize the variations in activation barrier heights across different substrates, and second, to uncover the underlying factors that drive the regioselectivity toward addition@C or addition@X.

**Scheme 1:**
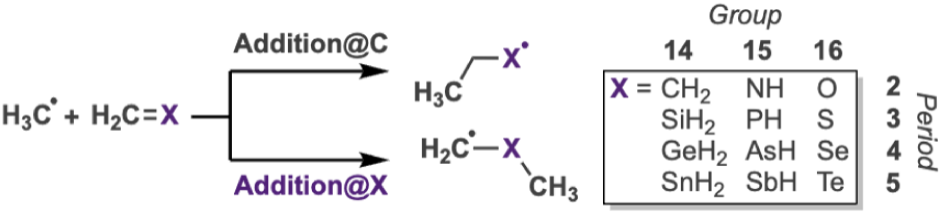
Studied Radical Addition Reactions (RAR)

The prevalent mechanistic framework of RARs is based on frontier molecular orbital (FMO) theory.^5^ In this approach the main interactions are posited to be orbital interactions between the singly occupied molecular orbital (SOMO) of the methyl radical with the highest occupied (HOMO) and lowest unoccupied (LUMO) molecular orbitals of the substrate (Scheme 2b). The strength of this interaction is determined by the energy gap (Δε) and overlap between the orbitals. The FMO framework predicts that a more electronegative X group (Scheme 2a) lowers the energies of the substrate HOMO and LUMO strengthening the SOMO–LUMO interaction. An electropositive X group (Scheme 2c) pushes the HOMO and LUMO of the substrate up in energy, leading to a strong SOMO–HOMO interaction. A more detailed explanation of Scheme 2 is provided in Section S1. The utility of FMO theory is derived from its very intuitive nature and its tolerance of a wide range of chemical transformations. However, FMO theory does not provide an immediately clear prediction of whether electropositive or electronegative X groups lead to stronger orbital interactions in RARs. This shortcoming of FMO theory is an outcropping of its neglect of Pauli repulsive (Δ*E*_Pauli_) interactions between key MOs. Consequently, the absence in accounting for these interactions results in inaccurate predictions of reactivity and selectivity. Recent studies have highlighted the importance of including Δ*E*_Pauli_ in understanding an array of reaction mechanisms.^6^

**Scheme 2:**
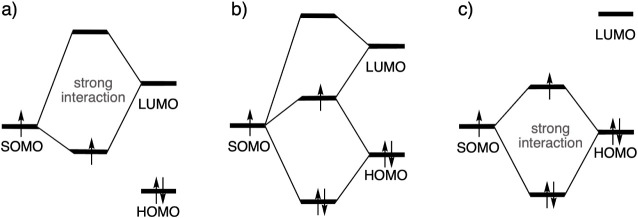
Frontier Molecular Orbital (FMO) Diagrams Highlighting the Effect of Substrate Substitutions on Substrate Frontier Orbitals: (a) Electronegative X Group, (b) Neutral X Group (e.g., CH_2_), (c) Electropositive X Group^a^

a See Section S1 for more details.

With the aim of providing a more unified picture of RARs, we employed relativistic density functional theory (DFT) at ZORA-(U)­OLYP/TZ2P using the Amsterdam Density Functional (ADF2024)^7^ program implemented in the Amsterdam Modeling Suite (AMS2024).^7^ In the past, DFT calculations by both Coote and Radom have been instrumental in accurately computing radical addition barriers and reaction energies, thereby enhancing our understanding of associated reactivity and selectivity trends.^8^ Within the framework of Kohn–Sham molecular orbital theory,^9^ the Activation Strain Model (ASM)^10^ and Energy Decomposition Analysis (EDA)^11^ were utilized to dissect the electronic energy contributions along the reaction coordinate into meaningful components. These tools provide a quantitative understanding of reactivity and regioselectivity, offering a detailed mechanistic picture of the factors governing these radical addition reactions. This approach aims to provide a fundamental explanation of established experimental observations and computational insights to assist in the rational design of regioselective radical transformations in synthetic chemistry.

## Results and Discussion

### General Trends in Reactivity

Studies commenced by examination of the reaction profiles for CH_3_^•^ + H_2_C=X (X = CH_2_, SiH_2_, GeH_2_, SnH_2_, NH, PH, AsH, SbH, O, S, Se, Te).^12^ Electronic activation barriers (Δ*E* ^‡^) and reaction energies (Δ*E*_rxn_), relative to the separate reactants, for both the addition@C and addition@X pathways are provided in Table 1. Structures along the PES of the methyl radical addition reactions of H_2_C=CH_2_, H_2_C=NH and H_2_C=O are shown in Figure 1. All optimized stationary points are provided in Table S3.

**Figure 1:**
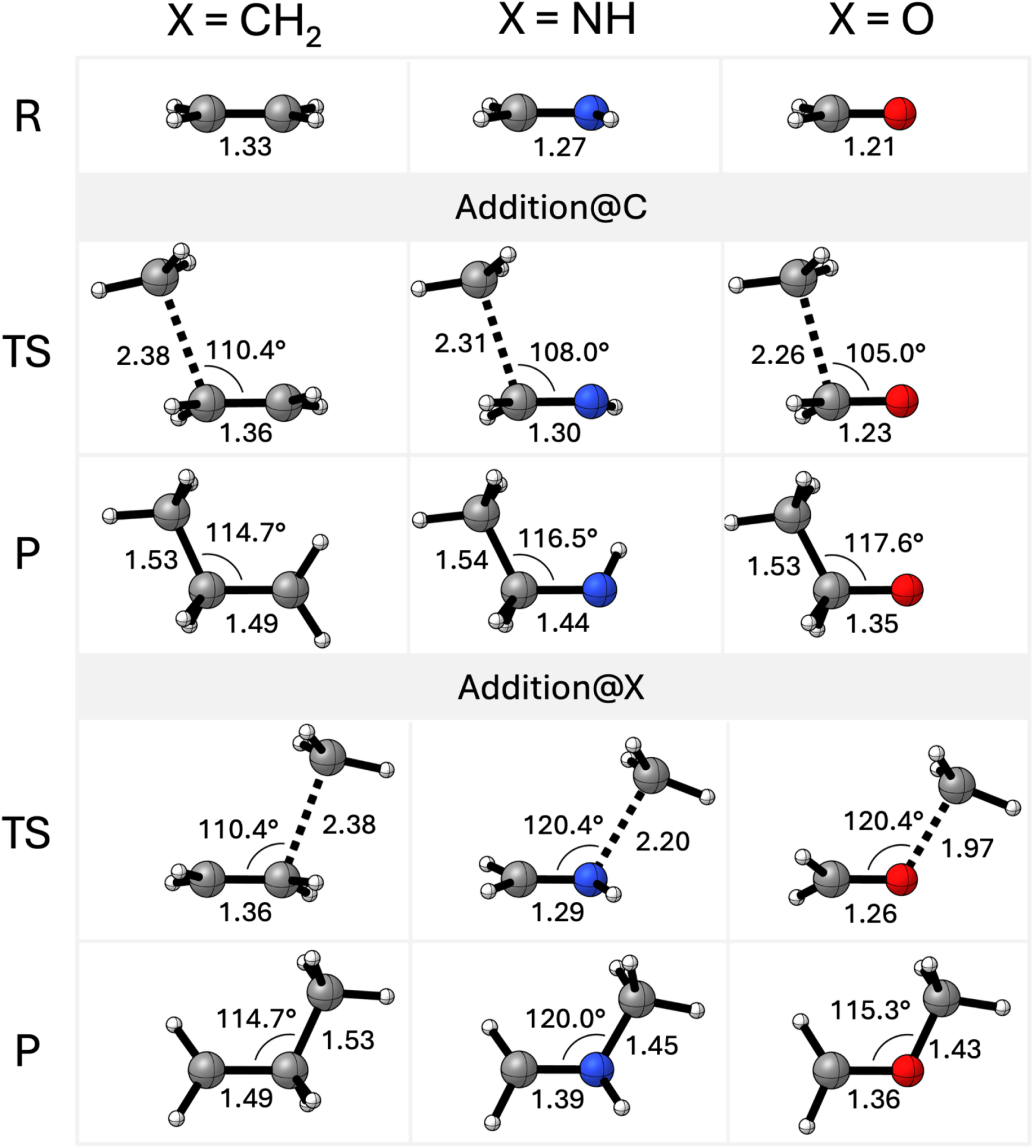
Optimized structures of addition@C and addition@X pathways for H_3_C^•^ addition to H_2_C=X (X = CH_2_, NH, and O) computed at ZORA-(U)­OLYP/TZ2P. Key bond distances (in Å) and angles (in °) are provided.

**Table 1:**
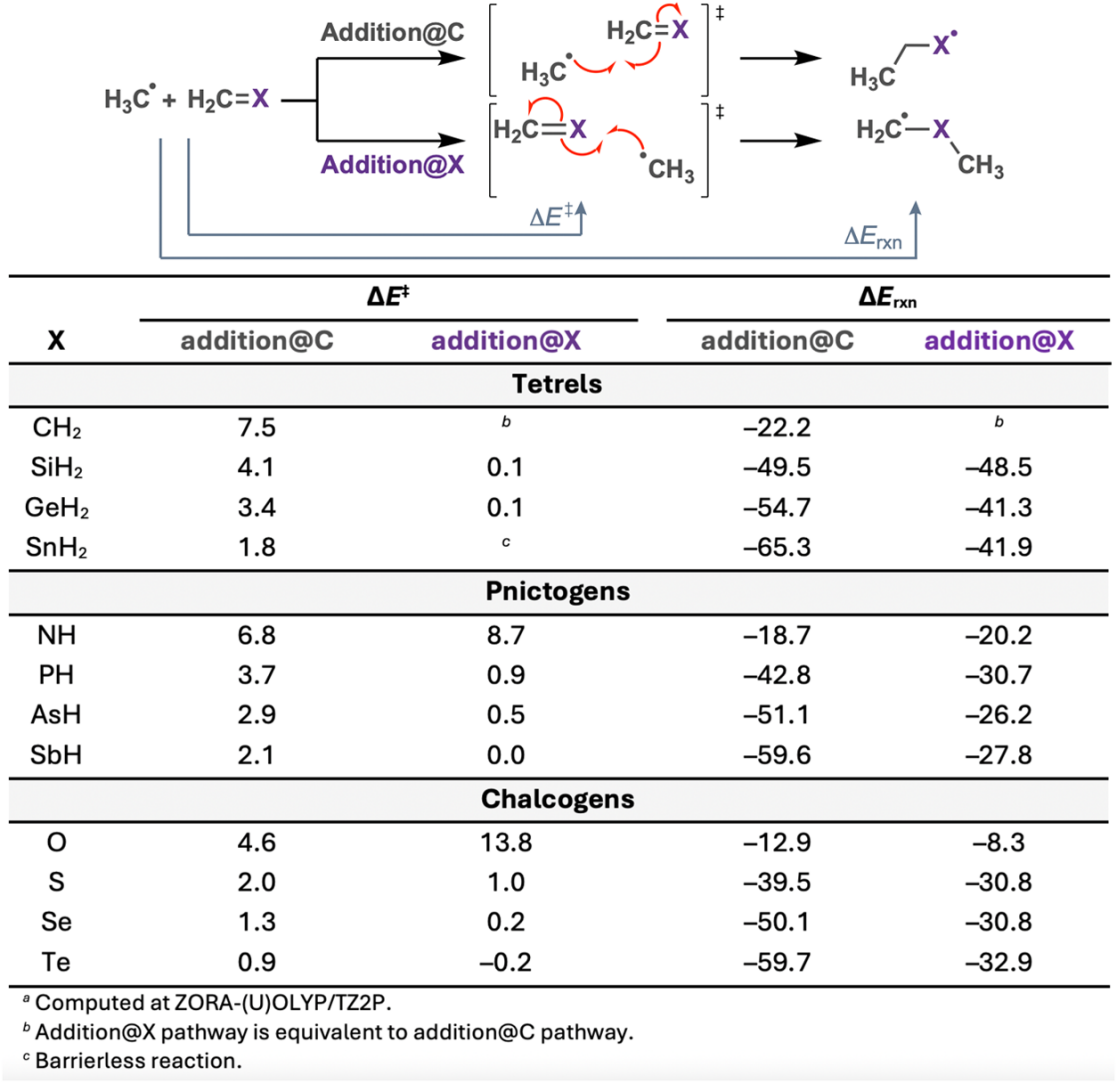
Activation Barriers (Δ*E* ^‡^) and Reaction Energies (Δ*E*_rxn_) (in kcal mol^–1^) for the Reaction of CH_3_^•^ + H_2_C=X via Addition@C and @X^a^

We uncovered three general trends from our reactivity analysis across the periodic table (*vide infra*). First, we investigate the effect of varying the X group of the substrate H_2_C=X from Group 14–16 along a Period. Activation barriers for addition@C decrease systematically as X transitions from tetrels (Group 14) to pnictogens (Group 15) to chalcogens (Group 16). For instance, the activation barriers (Δ*E* ^‡^) for addition@C drops from 7.5 (X = CH_2_) to 6.8 (X = NH) to 4.6 kcal mol^–1^ (X = O). Conversely, addition@X barriers increase along this series, rising from 7.5 (X = CH_2_) to 8.7 (X = NH) to 13.8 kcal mol^–1^ (X = O). Reaction energies (Δ*E*_rxn_) for both pathways become less favorable from Group 14 to Group 16, as observed for addition@C: −22.2 (X = CH_2_), −18.7 (X = NH), and −12.9 kcal mol^–1^ (X = O). Minor deviations from these trends, such as small variations in barriers for addition@X reactions within a group, were noted, but these do not disrupt the general pattern. Of note, the addition@X reaction for H_2_C=SnH_2_ is barrierless.

Second, we investigate the effect of varying the X group of the substrate H_2_C=X from Periods 2–5 along a Group. In general, both addition@C and @X barriers decrease (slightly) as X moves down a Group. For example, addition@C barriers for tetrels (Group 14) decrease from 7.5 (X = CH_2_) to 4.1 (X = SiH_2_), 3.4 (X = GeH_2_), and 1.8 kcal mol^–1^ (X = SnH_2_). This systematic trend also holds for pnictogens and chalcogens. Similarly, reaction energies for addition@C become more exothermic down the group, as seen for tetrels: −22.2 (X = CH_2_), −49.5 (X = SiH_2_), −54.7 (X = GeH_2_), and −65.3 kcal mol^–1^ (X = SnH_2_). Minor exceptions, such as slightly more favorable reaction energies for SiH_2_ and PH compared to their third-row counterparts, were observed.

Third, we compare addition@C and @X at the H_2_C=X substrate. In general, addition@X barriers are lower than their addition@C counterparts. For example, the addition@X barrier of H_2_C=PH is 0.9 kcal mol^–1^, significantly lower than 3.7 kcal mol^–1^ for addition@C. The exceptions are H_2_C=NH and H_2_C=O, which have more favorable addition@C barriers (6.8 and 4.6 kcal mol^–1^, respectively) compared to their addition@X barriers (8.7 and 13.8 kcal mol^–1^, respectively). Reaction energies for addition@C are generally more exothermic than those for addition@X, except for X = NH, where Δ*E*_rxn_ for addition@X (−20.2 kcal mol^–1^) is slightly more favorable than for addition@C (−18.7 kcal mol^–1^).

Of note, carbon-centered radical addition reactions to carbonyls (X = O), despite having a low activation barrier of 4.6 kcal mol^–1^ for addition@C, have not been reported in the literature thus far. This can be rationalized by a series of other competing reactions.^13^ Furthermore, the retro-barrier of 18.4 kcal mol^–1^ for addition@C is relatively low compared to those of X = CH_2_ (29.7 kcal mol^–1^) and NH (25.5 kcal mol^–1^), possibly leading to β-scission reactions.^14^ We observed that lower activation barriers correlate with more exothermic reaction energies for addition@C (Figure S1). However, the same correlation is not present for the addition@X reactions. Therefore, in the coming sections, we focus on the analysis of activation barriers of the addition@C and addition@X reactions.

In the next section, we highlight illustrative examples and focus on distinct periodic trends to provide a foundation for understanding the reactivity and regioselectivity of methyl radical addition to H_2_C = X substrates.

### Origin of Reactivity for Addition@C to H_2_C=X (X= CH_2_ to NH to O)

To elucidate the physical factors leading to the lower, more favorable addition@C activation barriers as X changes from CH_2_ to NH to O, we decompose the total electronic energy (Δ*E*) into the strain energy (Δ*E*_strain_) and the interaction energy (Δ*E*_int_). The activation strain analyses are provided in the top row of Figure 2 while the energy decomposition analyses are provided in the bottom row of Figure 2. The interacting fragments in the activation strain analysis are the methyl radical (H_3_C^•^) and H_2_C=X (X = CH_2_, NH, and O).

**Figure 2:**
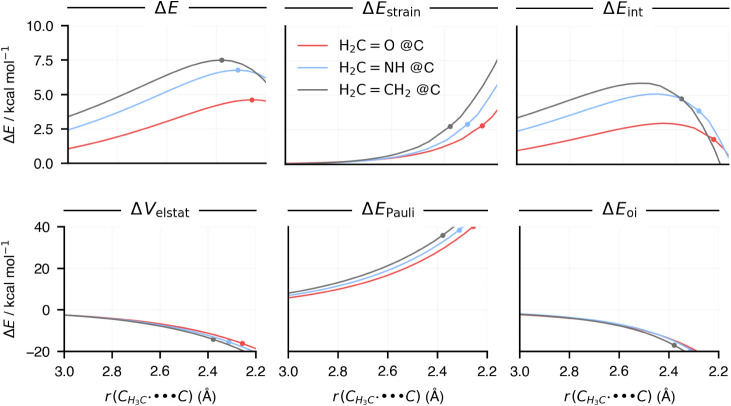
Activation strain model (top row) and energy decomposition analysis (bottom row) energy terms along the methyl radical–substrate distance of the reaction of H_2_C=X + CH_3_^•^ (X = CH_2_, NH, O) via the addition@C pathway. The dots represent the positions of the transition states of the respective reactions. Computed at ZORA-(U)­OLYP/TZ2P.

The more favorable addition@C activation barriers as X changes from CH_2_ to NH to O arise from a synergistic effect of reduced destabilizing strain energy and enhanced stabilizing interaction energy. To understand the origin of the strain energy trend, we decompose the total strain energy, Δ*E*_strain_, into contributions from the two reactants: the strain energy of the methyl radical, $\Delta E_{\mathrm{strain},H_{3}C^{•}}$ , and the strain energy of the substrate, $\Delta E_{\mathrm{strain},H_{2}C=X}$ , as shown in eq 2 (see Figure 3). Computed at ZORA-(U)­OLYP/TZ2P.

**Figure 3:**
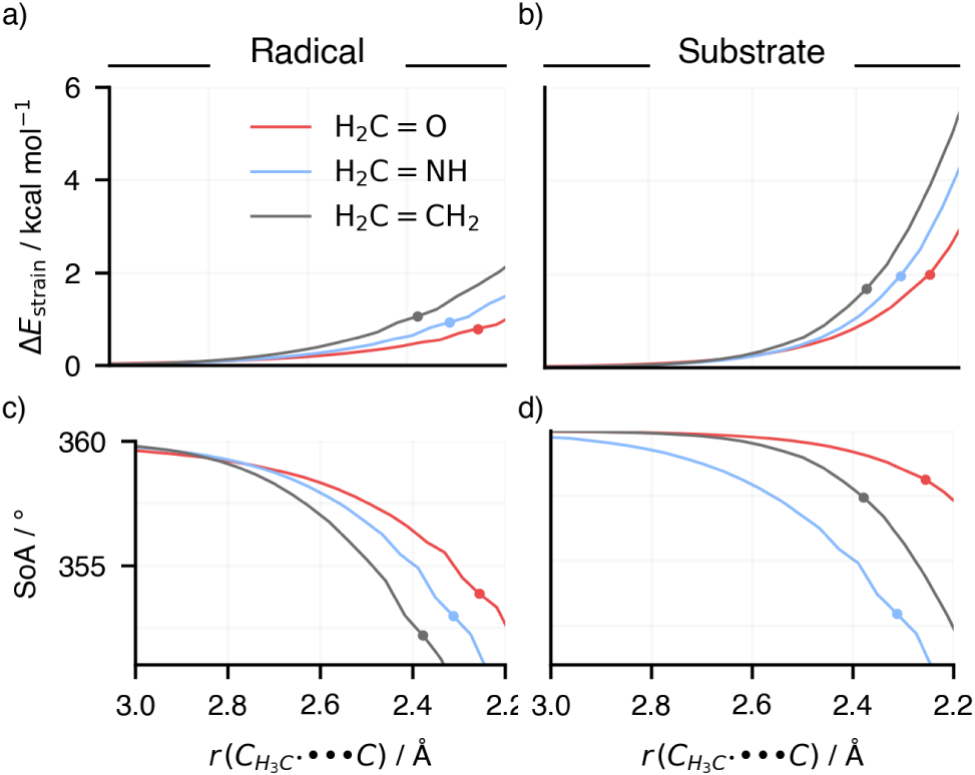
(a) Strain energy of the methyl radical and (b) substrate projected onto the radical–substrate reaction coordinate. The extent of pyramidalization of (c) the methyl radical and (d) the CH_2_ group of the substrate is measured with the sum-of-angles (SoA) around the central atom, which are the methyl carbon atom and the carbon atom of the substrate. A SoA of 360° indicates trigonal planar geometry. Computed at ZORA-(U)­OLYP/TZ2P.

Our analysis reveals that approximately a quarter of the strain energy originates from the methyl radical, with the remainder arising from the substrate. The primary distortion mode in these reactions involves the pyramidalization of both the radical and the substrate CH_2_ group (Figure 3c,d), which was measured by the sum of bond angles around the pyramidalizing atom. Notably, the extent of pyramidalization correlates with the strain energy, being highest for X = CH_2_ and lowest for X = O. This relationship holds for both radical and substrate strain and pyramidalization. These findings can be traced back to the bond strengths of the C=X double bond, which becomes progressively stronger as X changes from CH_2_ to NH to O. Increased pyramidalization weakens the double bond, reducing the overlap between the 2p orbitals of the bond-forming atoms of the X and CH_2_ groups (see Figure S2) for the effect of bond stretching on the overlap). Consequently, X = O exhibits less pyramidalization and lower strain energy, while X = CH_2_, with its weaker C=C bond, undergoes greater pyramidalization and higher strain energy. Additionally, the methyl radical pyramidalizes from an originally trigonal planar geometry in response to the substrate’s distortion, optimizing stabilization through interaction terms.

Next, to understand where the trend in more stabilizing interaction energy for the addition@C as X changes from CH_2_ to NH to O originates, we turn to the EDA to decompose Δ*E*_int_ into the repulsive Pauli repulsion term (Δ*E*_Pauli_), attractive orbital interaction (Δ*E*_oi_) and electrostatic interaction (Δ*V*_elstat_) (see Figure 2 bottom row). Our EDA results reveal that the trend in Δ*E*_int_ originates from Δ*E*_Pauli_. Both the Δ*V*_elstat_ and Δ*E*_oi_ terms follow the opposite trend from Δ*E*_int_ and, therefore, do not work to set the overall reactivity trends.

Steric Pauli repulsion arises from the unfavorable interaction between occupied orbitals, which leads to a mixing that destabilizes the system. The strength of this repulsion is primarily governed by the degree of overlap between the interacting orbitals. To better understand the origins of the trend in Δ*E*_Pauli_, we focus on the overlap between the π-HOMO of the substrate and the SOMO of the methyl radical (Figure 4). While other orbital pairs also contribute to Pauli repulsion, the π-HOMO/SOMO interaction exhibits the highest overlap and is therefore the most significant in determining the overall trend in steric repulsion. Our analysis reveals that the overlap increases consistently as the reactants approach one another for all three reactions. Importantly, the trend in orbital overlap mirrors that of Δ*E*_Pauli_, with the overlap decreasing systematically from X = CH_2_ to NH to O at equivalent geometries. This pattern reflects the influence of the electronic structure of the substrate, which changes as X varies. To gain deeper insight, we examined the orbital coefficients of the carbon atom and the heteroatom (X) within the substrate’s π-HOMO (Figure 4). We find that as X changes from CH_2_ to NH to O, the coefficient associated with the substrate’s carbon atom decreases. This reduction in the carbon coefficient leads to a diminished overlap with the SOMO of the methyl radical, ultimately contributing to the observed trend in Δ*E*_Pauli_. The origin of this behavior lies in the electronegativities of the X groups. As the electronegativity of X increases, the energy of the X group’s 2p orbital is lowered relative to the carbon 2p orbital (Figure 4). This energy shift causes the π-HOMO to polarize toward the X group, resulting in a larger orbital coefficient for the heteroatom and a correspondingly smaller coefficient for the carbon atom. This redistribution of orbital character reduces the extent of interaction between the substrate’s π-HOMO and the radical’s SOMO. Consequently, the overlap—and thus the steric Pauli repulsion—decreases along the series from X = CH_2_ to NH to O. These findings highlight the critical role of orbital composition and electronegativity in shaping the steric repulsion and provide a detailed explanation for the observed trends.

**Figure 4:**
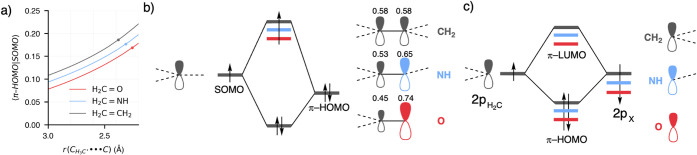
(a) Overlap between the π-HOMO of the substrate and the SOMO of the methyl radical along the methyl radical–substrate distance. The dots represent the positions of the transition states of the respective reactions. (b) The main repulsive orbital interaction between the SOMO of the methyl radical and the π-HOMO of the substrates responsible for setting the trend in the steric Pauli repulsion. The numbers above the π-HOMO show the coefficients of the atomic orbitals for the respective MOs. (c) Formation of the substrate π-HOMO and π-LUMO from the H_2_C and X fragments. Computed at ZORA-(U)­OLYP/TZ2P.

### Origin of Regioselectivity for Addition@C and @X to H_2_C=NH

Next, to understand the preference for either addition@C or addition@X, we performed a subsequent ASM and EDA analysis, comparing the energy terms for these two pathways on the H_2_C=NH substrate (Figure 5). We recall that addition@C goes with a lower activation barrier of 6.8 kcal mol^–1^ compared to addition@X 8.7 kcal mol^–1^. Importantly, the guiding reactivity principles we have uncovered from this reaction also hold for other reactions studied (Figure S3). Our analysis reveals that the preference for addition@C over addition@X is primarily driven by a more stabilizing interaction energy, as the trend in strain energy favors addition@X (Figure 5, top row). Diving deeper into the EDA results, we find that orbital interactions play an important role in setting the trend in interaction energy, with electrostatic interactions also contributing, but to a lesser extent (Figure 5, bottom row). These stabilizing orbital interactions are largely attributed to the interaction of the radical SOMO with the π-HOMO and π-LUMO orbitals of the substrate (Figure 6).

**Figure 5:**
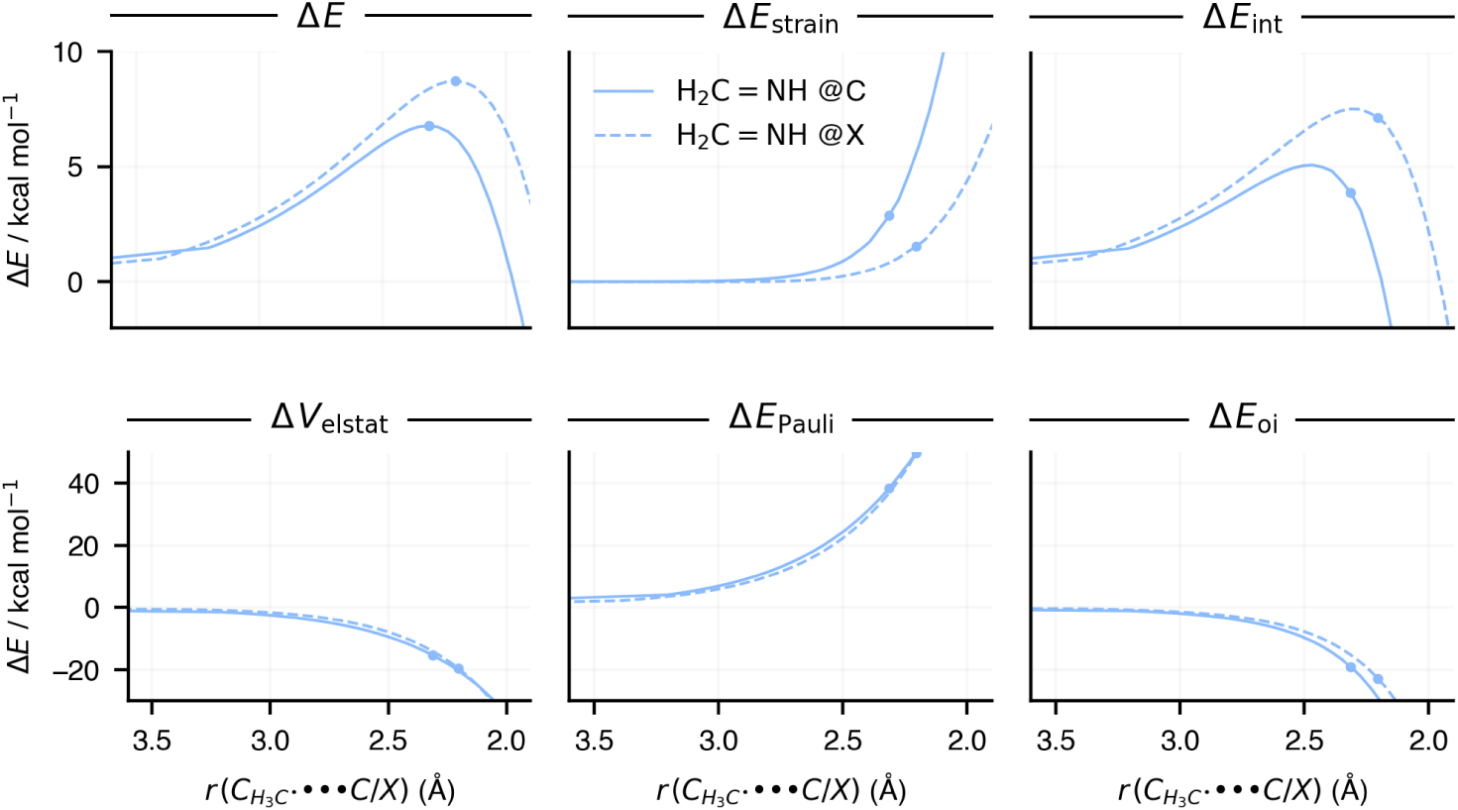
Activation strain model (top row) and energy decomposition analysis (bottom row) energy terms along the methyl radical–substrate distance of the reaction of H_2_C=NH + CH_3_^•^ via either the addition@C (solid lines) or @X (dashed lines) pathways. The dots represent the positions of the transition states of the respective reactions. Computed at ZORA-(U)­OLYP/TZ2P.

**Figure 6:**
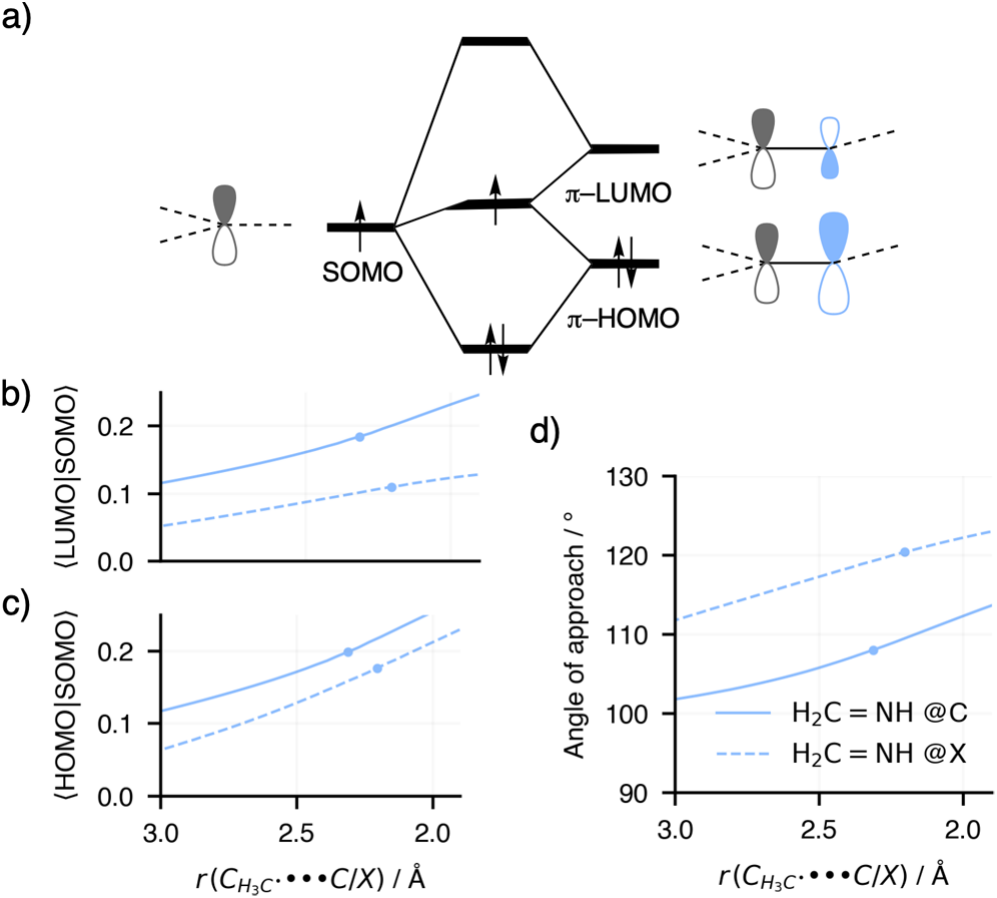
(a) Orbital interaction scheme for the reaction of CH_3_^•^ + H_2_C=NH via either the addition@C (solid lines) or @X (dashed lines) pathways. (b) Overlap between SOMO and π-LUMO and (c) overlap between SOMO and π-HOMO. (d) The angle of approach calculated as the N–C–C_radical_ angle for the addition@C pathway or the C–N–C_radical_ for the addition@X pathway. Computed at ZORA-(U)­OLYP/TZ2P.

In the case of the addition@C reaction, we observe that throughout the entire potential energy surface (PES), the overlaps between the SOMO and the π-HOMO, as well as between the SOMO and the π-LUMO, are consistently higher than those in the addition@X reaction. This greater overlap leads to stronger orbital interactions in the addition@C pathway, even though the orbital energy gaps between the interacting orbitals are nearly identical along both reaction pathways. The stabilization arising from orbital interactions can be quantitatively estimated using the second-order perturbation approximation,^9^b $\Delta E_{ij}^{\left(2\right)}\propto -\langle \psi_{i}|{\psi_{j}\rangle }^{2}/\left|\varepsilon_{i}-\varepsilon_{j}\right|$ , where ε*_i_* and ε*_j_* represent the orbital energies of ψ*_i_* (filled orbital) and ψ*_j_* (unfilled orbital), respectively. According to this approximation, the stabilization energy $\left(\Delta E_{ij}^{\left(2\right)}\right)$ is proportional to the square of the orbital overlap $\left(\langle \psi_{i}|{\psi_{j}\rangle }^{2}\right)$ and inversely proportional to the energy gap $\left(\left|\varepsilon_{i}-\varepsilon_{j}\right|\right)$ ). Applying this approximation to our system, we find that the increased overlap between the SOMO and the substrate’s orbitals in the addition@C pathway results in significantly greater stabilization compared to the addition@X pathway. This enhanced orbital interaction energy explains why addition@C is energetically favored over addition@X. These findings underscore the importance of orbital alignment and overlap in determining reaction preferences, highlighting how even small differences in overlap can strongly influence the energetics of competing pathways.

The increased overlap observed in the addition@C pathway can be attributed to the influence of the nitrogen atom in the H_2_C=NH substrate. As a more electronegative element, nitrogen polarizes the π-HOMO toward itself, resulting in a larger coefficient on the nitrogen 2p orbital. In principle, this polarization should enhance the overlap between the radical SOMO and the substrate’s π-HOMO in the addition@X pathway, where the radical approaches the nitrogen atom. However, this expected increase in overlap is counteracted by the geometry of the addition@X pathway.

Specifically, in the addition@X pathway, the radical approaches the substrate at a larger angle, which reduces the alignment between the interacting orbitals. Reduced alignment decreases the overlap between the SOMO and the π-HOMO, thereby weakening the orbital interaction. In contrast, the addition@C pathway benefits from a more favorable approach angle. The smaller angle allows for better orbital alignment, enhancing the overlap and stabilizing the interaction. Additionally, the π-LUMO of the H_2_C=NH substrate is polarized toward the carbon atom due to the presence of the nitrogen atom. This polarization increases the coefficient on the carbon 2p orbital, thereby improving the overlap with the radical SOMO during the addition@C pathway. The already favorable overlap is further reinforced by the smaller angle of approach of the radical in this pathway, which optimizes the alignment of the interacting orbitals.

Together, these factors—the polarization of the π-LUMO toward the carbon atom, the smaller angle of approach, and the improved orbital alignment—contribute to the increased overlap and stronger orbital interactions in the addition@C pathway. This enhanced overlap not only stabilizes the addition@C pathway but also explains its preference over the addition@X pathway.

### Origin of Regioselectivity for Addition@C and @X to H_2_C=PH

While for Period 2 elements (e.g., C, O and N) the addition@C is preferred over addition@X, this preference is reversed in the case of Period 3, 4, and 5 elements. Similarly to the Period 2 elements, ASM and EDA analyses reveal that it is the orbital interactions that determine the preference for the addition@C pathway (see Figure 7 for H_2_C=PH and Figure S4 for H_2_C=S). In the case of H_2_C=PH, we find that specifically the orbital overlap between the substrate’s π-LUMO and the radical SOMO is higher via the addition@X pathway (see Figure 8). This is because the 3p valence orbitals of P are larger in size than the 2p valence orbitals of the Period 2 elements, leading to a polarization of the π-LUMO toward the X group. The larger overlap leads to stronger orbital interactions, in turn leading to more favorable activation barriers. The overlap for the addition@X is larger despite a larger angle-of-approach, which decreases the overlap. These findings reinforce that the polarization of the substrate’s molecular orbitals is critical in determining the regioselectivity of the methyl radical addition reaction.

**Figure 7:**
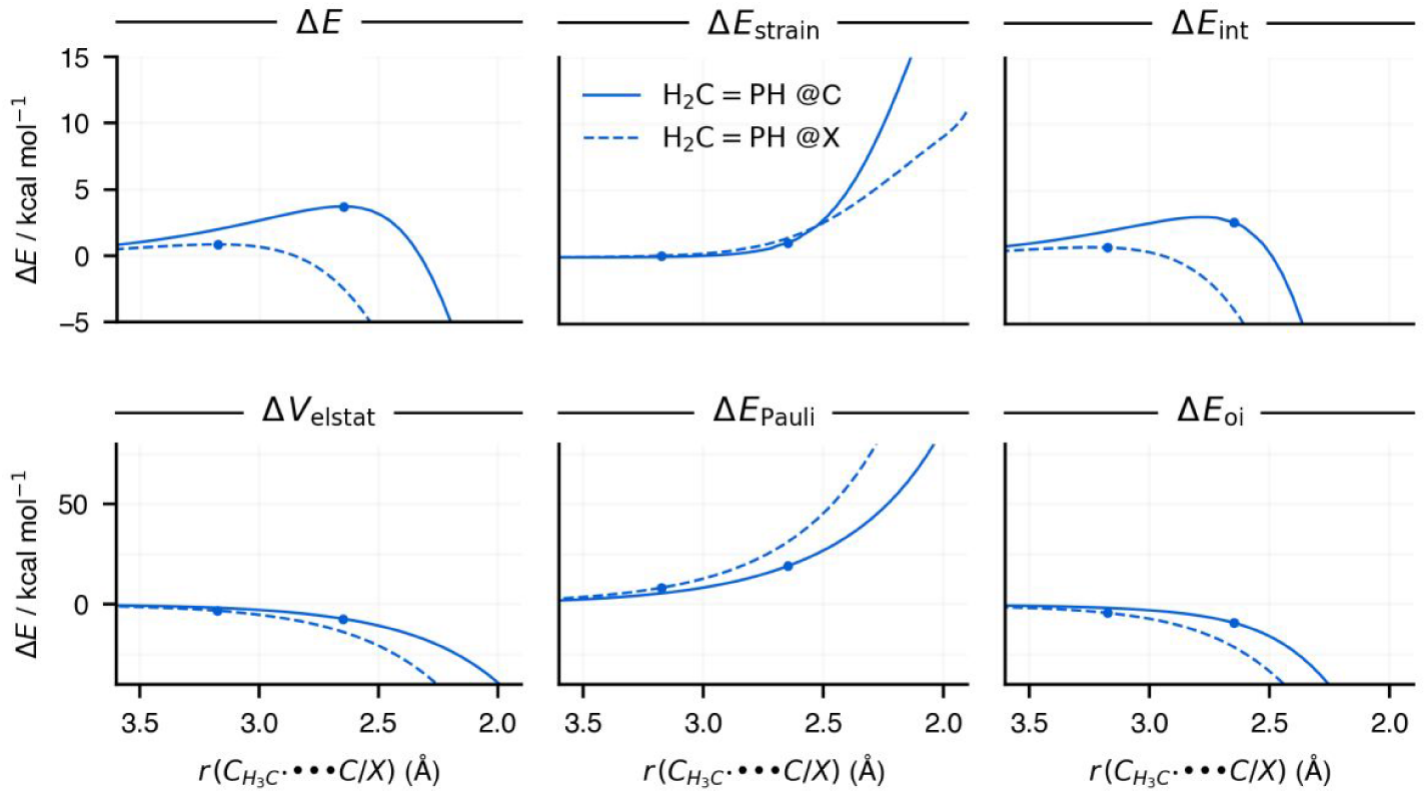
Activation strain model (top row) and energy decomposition analysis (bottom row) energy terms along the methyl radical–substrate distance of the reaction of H_2_C=PH + CH_3_^•^ via either the addition@C (solid lines) or @X (dashed lines) pathways. The dots represent the positions of the transition states of the respective reactions. Computed at ZORA-(U)­OLYP/TZ2P.

**Figure 8:**
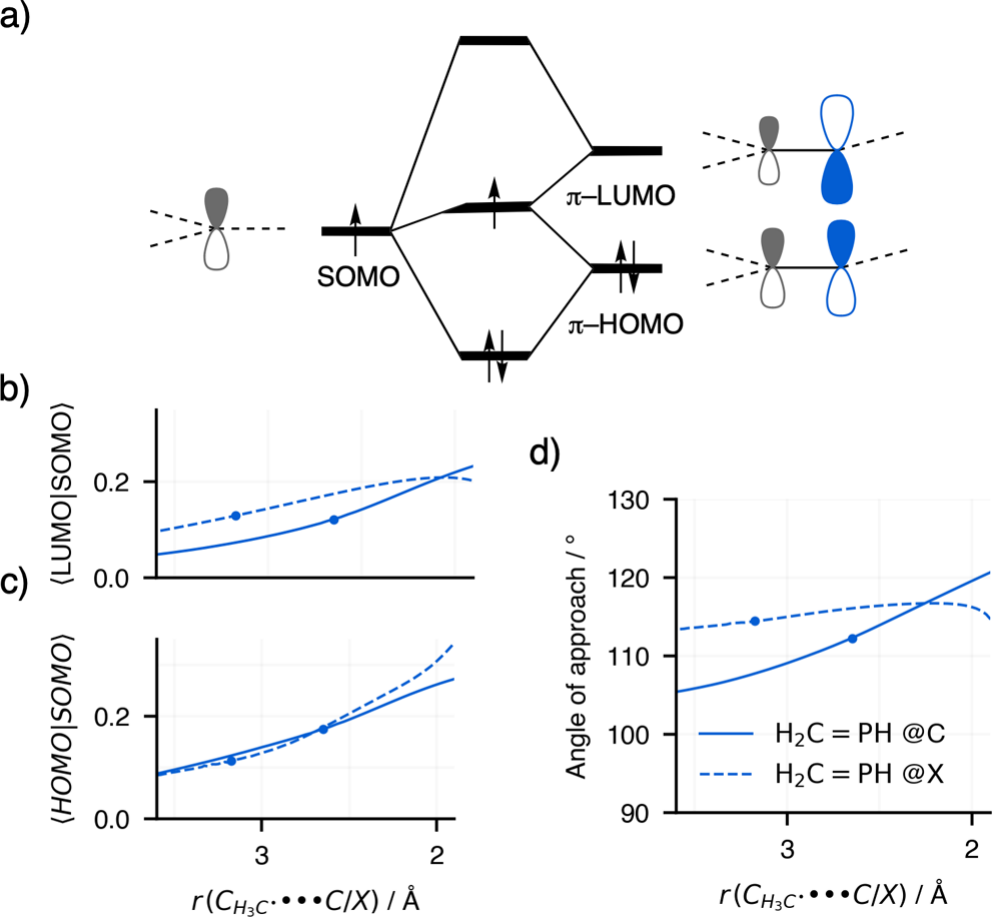
(a) Orbital interaction scheme for the reaction of H_2_C=PH + CH_3_^•^ via either the addition@C (solid lines) or @X (dashed lines) pathways. (b) Overlap between SOMO and π-LUMO and (c) overlap between SOMO and π-HOMO. (d) The angle of approach is calculated as the P–C–C_radical_ angle for the addition@C pathway or the C–P–C_radical_ for the addition@X pathway. Computed at ZORA-(U)­OLYP/TZ2P.

For the benefit of applications such as machine learning, we further performed static ASM and EDA analyses on the transition state geometries of our reactions (see Section S2).

## Conclusions

In this study, we have systematically investigated the methyl radical addition reactions on H_2_C=X substrates, where X is varied across elements from the tetrel (group 14: C, Si, Ge), pnictogen (group 15: N, P, As), and chalcogen (group 16: O, S, Se) groups. Employing high-level ZORA-(U)­OLYP/TZ2P computations, we uncovered distinct trends in activation and reaction energies for both addition@C and @X pathways. Specifically, activation barriers for the addition@C pathway decrease as X progresses from tetrels to pnictogens to chalcogens, while the addition@X barriers exhibit the opposite trend, increasing with the same progression. Reaction energies for both pathways become less favorable across a period, whereas both activation barriers and reaction energies decrease down a group. Notably, the addition@X pathway is generally preferred, except for specific cases where X = CH_2_, NH, and O, where the addition@C pathway is favored. These insights behind the regioselectivity of methyl radical addition reactions on H_2_C=X substrates are summarized in Scheme 3.

**Scheme 3:**
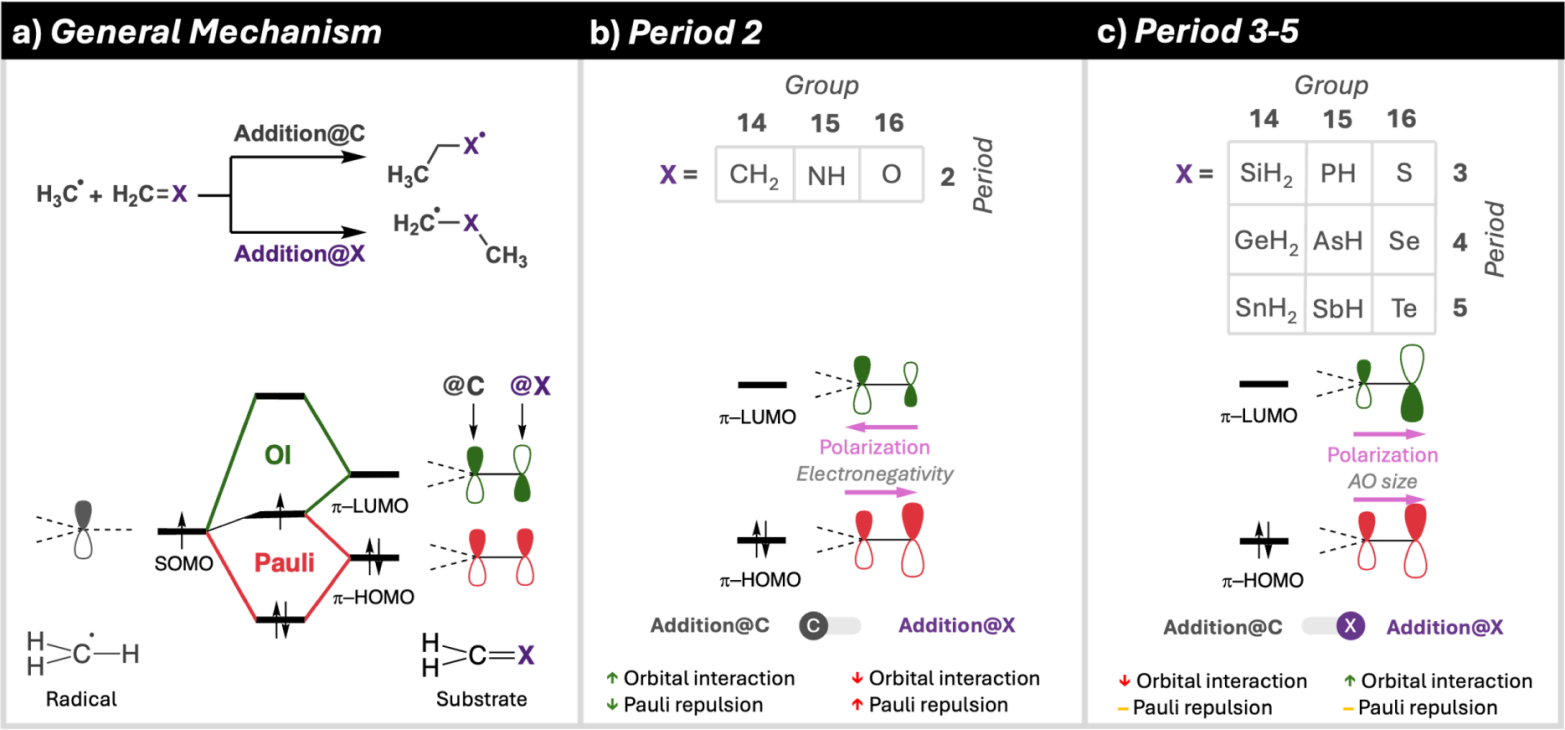
Summary of Guiding Features Controlling the Regioselectivity of Methyl Radical Addition Reactions on H_2_C=X Substrates

Through detailed Activation Strain Model (ASM) and Energy Decomposition Analysis (EDA), we identified the underlying physical origins of these trends. The preference for addition@C in cases such as X = CH_2_, NH, and O arises from a balance between strain and interaction energies. The strain energy trend correlates with the extent of pyramidalization of the radical and substrate, which varies with the electronic properties of X. The interaction energy is dominated by the Pauli repulsion term, which decreases as X becomes more electronegative, polarizing the π-HOMO away from the reactive H_2_C-group and reducing orbital overlap with the radical SOMO. Further ASM/EDA investigations elucidated the preference for the addition@C pathway over the addition@X pathway for Period 2 elements. The key factor is the orbital interaction between the radical SOMO and the substrate’s π-HOMO and π-LUMO. The addition@C pathway benefits from a more favorable overlap due to a smaller angle of approach, optimizing orbital alignment and stabilizing the reaction. For Period 3–5 elements the addition@X is preferred over the addition@C. Orbital interactions are still the determining factor, however the larger valence orbitals of the Period 3–5 elements compared to the valence orbitals of the Period 2 elements causes the π-LUMO to polarize toward the X group, leading to a preference for the addition@X pathway.

## Experimental Section

### Computational Methods

All calculations were performed using the generalized gradient approximation (GGA) functional OLYP,^15^ which consists of the optimized exchange (OPTX) functional proposed by Handy and Cohen,^15^a and the Lee–Yang–Parr (LYP) correlation functional^15^b was used for the optimizations of all stationary points, as well as for the analyses along the reaction coordinate, using the activation strain model (ASM) and energy decomposition analysis (EDA).^10,11^ The basis set employed for all atoms, denoted, TZ2P, is of triple-ζ quality and augmented with two sets of polarization functions.^16^ The radical fragments were treated with a spin-unrestricted formalism. Scalar relativistic effects were accounted for using the zeroth-order regular approximation (ZORA).^17^ Taken altogether, the computational level is ZORA-(U)­OLYP/TZ2P. Recently, we have shown the excellent performance of this functional in reproducing radical addition barriers and reaction energies computed at QRO–CCSD­(T)/CBS+.^18^ The accuracies of the integration grid (Becke grid)^19^a and the fit scheme (Zlm fit)^19^b were set to VERYGOOD. All geometries were optimized without any symmetry constraint. All stationary points were verified by vibrational analysis,^20^ confirming that equilibrium geometries contained no imaginary frequencies, and transition states contained one imaginary frequency. Additionally, the character of the normal mode associated with the imaginary frequency has been analyzed to ensure it was associated with the proper reaction mode: the formation of the C–C or C–X bond between the methyl radical and the substrate. The potential energy surfaces of the studied radical addition reactions were obtained through the performance of intrinsic reaction coordinate (IRC) calculations.^21^ The acquired potential energy surfaces were analyzed using the PyFrag 2019 program.^22^

### Activation Strain Model

The activation strain model of chemical reactivity (ASM, or distortion/interaction model^23^ is a fragment-based approach built upon the energy of a reacting system (i.e., the potential energy surface), which is described with respect to and understood in terms of the characteristics of the original reactants. The model examines the rigidity of the original reactants, the extent of their deformation during the reaction, and their ability to interact as the reaction proceeds. In this model, the potential energy surface Δ*E*(ζ) is decomposed into the total strain Δ*E*_strain_(ζ) of the reactants and interaction energy Δ*E*_int_(ζ) between the reactants in which these values are projected on the reaction coordinate ζ [eq 1].

1 $$\Delta E\left(\zeta \right)=\Delta E_{\mathrm{strain}}\left(\zeta \right)+\Delta E_{\mathrm{int}}\left(\zeta \right)$$

In this framework, the total strain energy Δ*E*_strain_(ζ) is the energy penalty required to deform the reactants from their equilibrium geometry, into the configuration they adopt during the reaction at position ζ of the reaction coordinate. The interaction energy Δ*E*_int_(ζ) accounts for all chemical interactions that arise between the deformed fragments along the reaction coordinate. The total strain energy can be further decomposed into the strain energies corresponding to the deformation of the two reactants, that is the methyl radical $\Delta E_{\mathrm{strain},H_{3}C^{•}}\left(\zeta \right)$ and the deformation of the substrate $\Delta E_{\mathrm{strain},H_{2}C=X}\left(\zeta \right)$ [eq 2]:

2 $$\Delta E_{\mathrm{strain}}\left(\zeta \right)=\Delta E_{\mathrm{strain},H_{3}C^{•}}\left(\zeta \right)+\Delta E_{\mathrm{strain},H_{2}C=X}\left(\zeta \right)$$

### Energy Decomposition Analysis

The interaction energy between the deformed reactants, the fragments, is further analyzed in terms of quantitative Kohn–Sham molecular orbital theory (KS-MO) in combination with a canonical energy decomposition analysis (EDA). In the EDA scheme, the Δ*E*_int_(ζ) is decomposed into the following physically meaningful energy terms [eq 3]:

3 $$\Delta E_{\mathrm{int}}\left(\zeta \right)=\Delta V_{\mathrm{elstat}}\left(\zeta \right)+\Delta E_{\mathrm{Pauli}}\left(\zeta \right)+\Delta E_{\mathrm{oi}}\left(\zeta \right)$$

Herein, Δ*V*_elstat_(ζ) is the classical electrostatic interaction between the unperturbed charge distributions of the deformed reactants and it is usually attractive. The Pauli repulsion Δ*E*_Pauli_(ζ) accounts for the destabilizing interaction between occupied closed-shell orbitals of both deformed reactants due to the Pauli principle. The orbital interaction energy Δ*E*_oi_(ζ) accounts for charge transfer and polarization between the fragments. A detailed step-by-step, guide on how to perform and interpret ASM and EDA can be found in ref. 10a.

The activation strain and energy decomposition analyses were carried out along the intrinsic reaction coordinate (IRC) and projected onto the most critical geometry parameter in this reaction, which is the forming $C_{H_{3}C^{•}}\cdot \cdot \cdot C$ or $C_{H_{3}C^{•}}\cdot \cdot \cdot X$ bond between the methyl radical (H_3_C^•^) and the substrate (H_2_C=X). This particular reaction coordinate undergoes a well-defined change that is intimately tied to the progress of the reaction.

## Supplementary Material



## Data Availability

The data underlying this study are available in the published article and its Supporting Information.

## Notes

Plot of activation barrier (Δ*E*‡) versus reaction energy (Δ*E*_rxn_) for the radical addition@C reactions of the chalcogens, pnictogens, and tetrels; overlap between the 2p_y_ atomic orbitals of the bond-forming atoms of the H_2_C=X double bond as a function of the double bond length; activation strain model and energy decomposition analysis energy terms for the reaction of H_2_C=O + CH_3_^•^; frontier molecular orbital interaction diagrams for the reaction of the radical’s SOMO with the substrate’s HOMO and LUMO split into alpha and beta spin contributions; ASM and EDA energy terms for the activation barriers and reaction energies of the addition@C and addition@X pathways; Cartesian coordinates (Å), electronic energies (*E*), enthalpies (*H*), Gibb’s free energies (*G*) (calculated at 298 K) and imaginary frequencies (*N*_imag_, only for transition states) for all stationary points (PDF)
