# Supplementary material for: Methyl Radical Addition Reactions to C=X Double Bonds
Source: J Org Chem. 2025 Jul 21;90(30):10786–96. doi: 10.1021/acs.joc.5c01157 (PMC12322914; doi:10.1021/acs.joc.5c01157)
Supplement: Supplementary file 1 [file jo5c01157_si_001.pdf]

Methyl Radical Addition Reactions to C=X Double Bonds

Yuman Hordijk,† Bart Waaijer,† Christopher B. Kelly,‡ Trevor A. Hamlin* †

† Department of Chemistry and Pharmaceutical Sciences, Amsterdam Institute for Molecular and Life Sciences (AIMMS), Vrije Universiteit Amsterdam De Boelelaan 1108, Amsterdam, 1081 HZ, The Netherlands

‡ Discovery Process Research, Johnson & Johnson Innovative Medicine, Spring House, Pennsylvania 19477, United States

Contents

Figure S1. Plot of activation barrier ($\Delta E^{‡}$) versus reaction energy (ΔE_{rxn}) for the radical addition@C reactions of the chalcogens, pnictogens, and tetrels. Linear regression equations and corresponding correlation coefficients are provided. All data calculated at ZORA-(U)OLYP/TZ2P. S2

Figure S2. Overlap between the $2p_y$ atomic orbitals of the bond-forming atoms of the $H_2C=X$ double bond as a function of the double bond length. All data calculated at ZORA-(U)OLYP/TZ2P. S2

Figure S3. Activation strain model (top row) and energy decomposition analysis (bottom row) energy terms along the methyl radical-substrate distance of the reaction of $H_2C=O + CH_3^{\bullet}$ (red lines) and $H_2C=NH + CH_3^{\bullet}$ (blue lines) via either the addition@C (solid lines) or addition@X (dashed lines) pathways. The dots represent the positions of the transition states of the respective reactions. All data computed at ZORA-(U)OLYP/TZ2P. S3

Figure S4. Activation strain model (top row) and energy decomposition analysis (bottom row) energy terms along the methyl radical-substrate distance of the reaction of $H_2C=S + CH_3^{\bullet}$ via either the addition@C (solid lines) or addition@X (dashed lines) pathways. The dots represent the positions of the transition states of the respective reactions. All data computed at ZORA-(U)OLYP/TZ2P. S4

Figure S5. Frontier molecular orbital interaction diagrams for the reaction of the radical's SOMO with the substrate's HOMO and LUMO split into alpha and beta spin contributions. The substrate was substituted with an electronegative, electroneutral, or electropositive substituent. S6

Table S1. ASM and EDA energy terms (in kcal mol^{-1}) for the activation barriers of the addition@C and addition@X pathways. S7

Table S2. ASM and EDA energy terms (in kcal mol^{-1}) for the reaction energies of the addition@C and addition@X pathways. S8

Table S3. Cartesian coordinates (Å), electronic energies (*E*), enthalpies (*H*), Gibb's free energies (*G*) (calculated at 298 K) and imaginary frequencies (N_{imag}, only for transition states) for all stationary points of the reaction of + $CH_3^{\bullet}$ + $H_2C=X$ via both Addition@C and Addition@X calculated at ZORA-(U)OLYP/TZ2P. S10

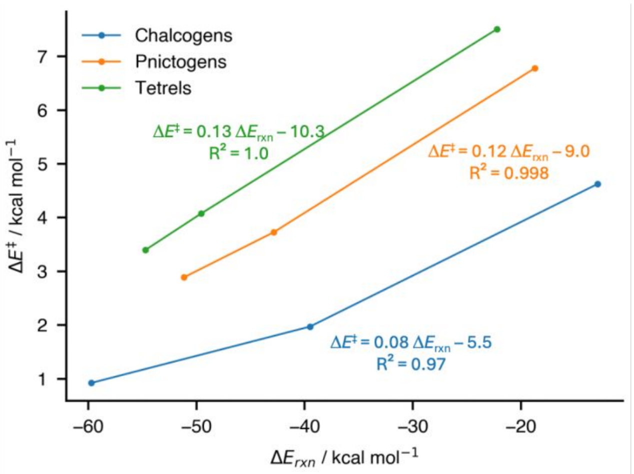

Figure S1. Plot of activation barrier ($\Delta E^{\ddagger}$) versus reaction energy (ΔE_{rxn}) for the radical addition@C reactions of the chalcogens, pnictogens, and tetrels. Linear regression equations and corresponding correlation coefficients are provided. All data calculated at ZORA-(U)OLYP/TZ2P.

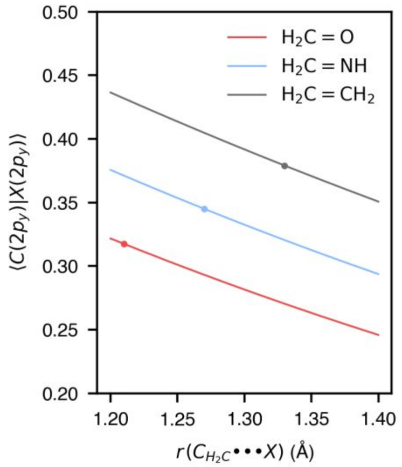

Figure S2. Overlap between the $2p_y$ atomic orbitals of the bond-forming atoms of the H_2C=X double bond as a function of the double bond length. All data calculated at ZORA-(U)OLYP/TZ2P.

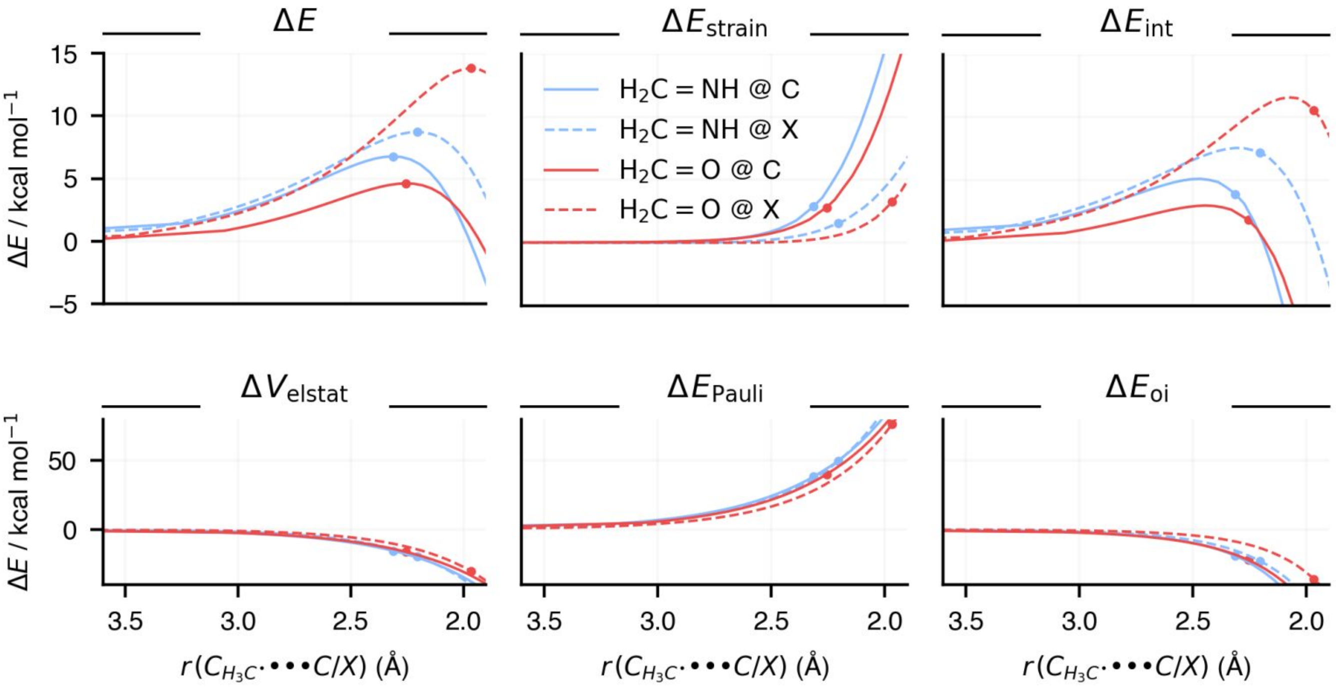

Figure S3. Activation strain model (top row) and energy decomposition analysis (bottom row) energy terms along the methyl radical-substrate distance of the reaction of $H_2C{=}O + CH_3^{\bullet}$ (red lines) and $H_2C{=}NH + CH_3^{\bullet}$ (blue lines) via either the addition@C (solid lines) or addition@X (dashed lines) pathways. The dots represent the positions of the transition states of the respective reactions. All data computed at ZORA-(U)OLYP/TZ2P.

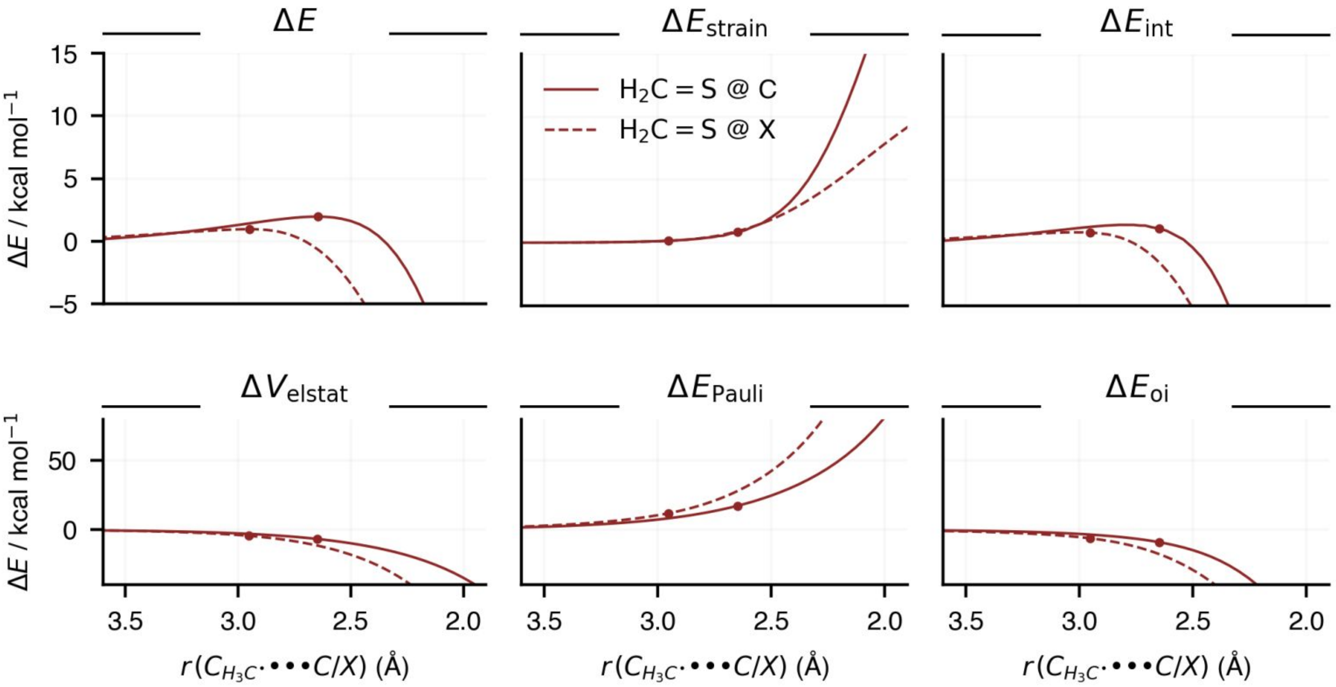

Figure S4. Activation strain model (top row) and energy decomposition analysis (bottom row) energy terms along the methyl radical-substrate distance of the reaction of $H_2C{=}S$ + $CH_3^{\bullet}$ via either the addition@C (solid lines) or addition@X (dashed lines) pathways. The dots represent the positions of the transition states of the respective reactions. All data computed at ZORA-(U)OLYP/TZ2P.

Section S1: Clarification on the Frontier Molecular Orbital Diagram in Scheme 2

In Scheme 2 we showed FMO diagrams for odd-electron orbital interactions between the radical's SOMO with the HOMO and LUMO of a substrate with electronegative, electroneutral, or electropositive substituents. We note that for, respectively, electronegative and electropositive substituents the SOMO/LUMO and SOMO/HOMO interactions are stabilizing. In the case of the electronegative substituent, we have a one electron-two center interaction, which should be clearly stabilizing. However, the stabilization of the three electron-two center interaction in the case of the electropositive substituent is less clear. The interactions can be clearly explained due to our use of unrestricted DFT methodology for calculations of radical species. This means that alpha and beta spin orbitals are treated separately, allowing us to analyze the interactions in each spin state separately.

Electronegative substituent

The interaction between the SOMO and the HOMO is weak due to the large energy gap, meaning that the main interaction is between the SOMO and LUMO. Due to the unrestricted nature of our calculation the SOMO/LUMO interaction is split into a stabilizing filled/unfilled interaction in the alpha spin and an inert unfilled/unfilled interaction in the beta spin, leading to an overall stabilizing interaction.

Electroneutral substituent

Both SOMO/HOMO and SOMO/LUMO interactions can be significant in the case of an electroneutral substituent. The interaction is split into a primarily stabilizing interaction in the beta spin and a stabilizing (SOMO/LUMO) and destabilizing (SOMO/HOMO) interaction in the alpha spin. This interaction therefore has stabilizing and destabilizing character.

Electropositive substituent

The main interaction is the SOMO/HOMO interaction as the LUMO is significantly higher in energy than the HOMO. The interaction can be split into a destabilizing filled/filled interaction in the alpha spin and a stabilizing filled/unfilled interaction in the beta spin. The stabilizing interaction is stronger than in the electroneutral case as the energy of the HOMO is raised to be closer to the LUMO when an electropositive substituent is used. This means that this interaction is overall more stabilizing than the electroneutral case.

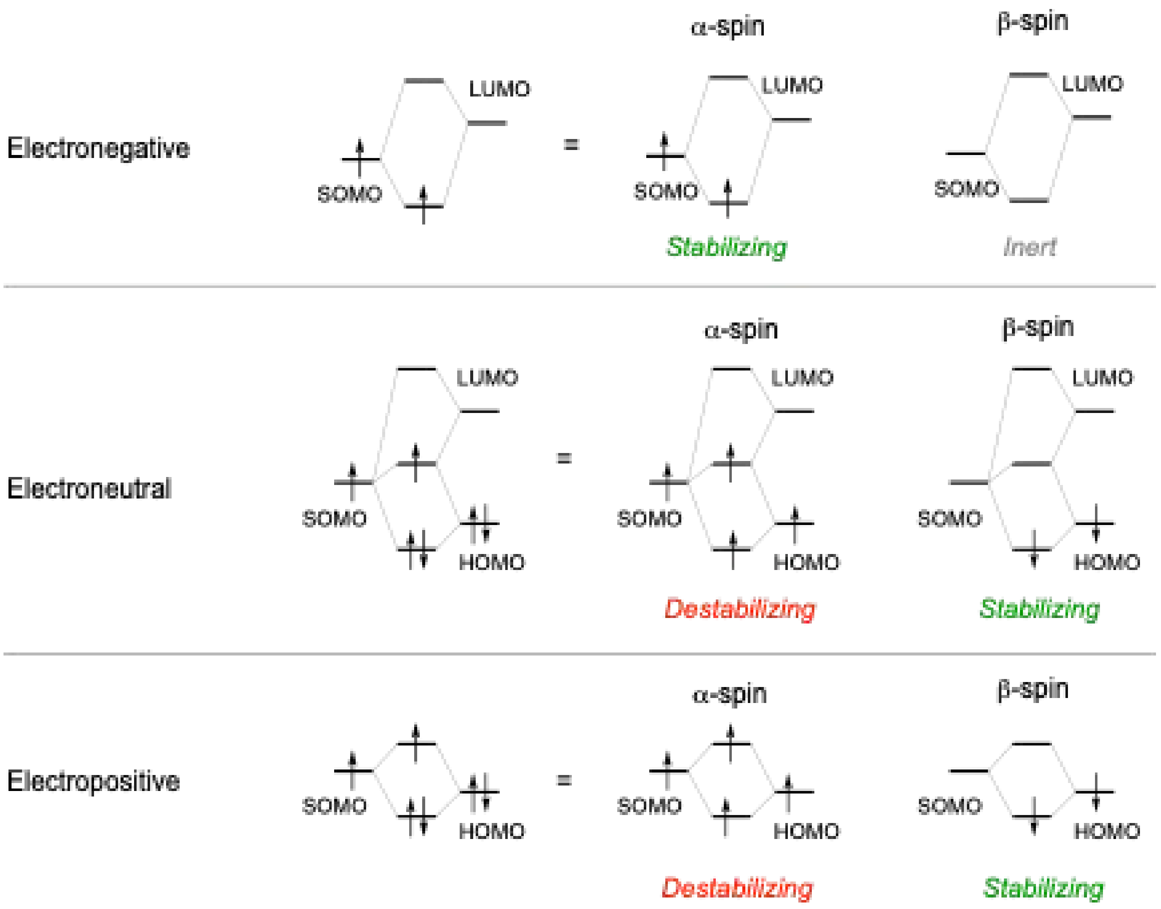

Figure S5. Frontier molecular orbital interaction diagrams for the reaction of the radical's SOMO with the substrate's HOMO and LUMO split into alpha and beta spin contributions. The substrate was substituted with an electronegative, electroneutral, or electropositive substituent.

Section S2: ASM/EDA Analyses on Transition State Geometries

The ASM/EDA analyses described in the main text were performed along the reaction coordinate and provide rich and detailed insights into the bond-forming process that drives the reaction. While this dynamic view reveals the underlying physical forces influencing the reaction, there are instances, such as predicting activation barriers using machine learning, where a static analysis of the transition state geometries is beneficial. To complement our previous findings, we have performed ASM/EDA analyses on the transition state geometries for both the addition@C and addition@X pathways (Table S1). For values at the product geometries, refer to Table S2.

Table S1. ASM and EDA energy terms (in kcal mol^{-1}) for the activation barriers of the addition@C and addition@X pathways.[a]

	Addition@C						Addition@X					
X	$\Delta E^{\ddagger}$	$\Delta E^{\ddagger}_{int}$	$\Delta E^{\ddagger}_{oi}$	$\Delta E^{\ddagger}_{Pauli}$	$\Delta V^{\ddagger}_{elstat}$	$\Delta E^{\ddagger}_{strain}$	$\Delta E^{\ddagger}$	$\Delta E^{\ddagger}_{int}$	$\Delta E^{\ddagger}_{oi}$	$\Delta E^{\ddagger}_{Pauli}$	$\Delta V^{\ddagger}_{elstat}$	$\Delta E^{\ddagger}_{strain}$
**Tetrels**												
CH_2	7.5	4.8	–17.0	35.9	–14.1	2.7	7.5	4.8	–17.0	35.9	–14.1	2.7
SiH_2	4.1	2.8	–7.8	16.4	–5.8	1.3	0.1	–0.0	–1.7	3.1	–1.4	0.1
GeH_2	3.4	2.2	–6.7	13.4	–4.5	1.2	0.1	–0.1	–1.9	3.6	–1.7	0.1
SnH_2	1.8	1.2	–3.8	7.0	–2.0	0.6				[b]		
**Pnictogens**												
NH	6.8	3.9	–19.1	38.4	–15.4	2.9	8.7	7.2	–22.9	49.6	–19.6	1.5
PH	3.7	2.6	–9.2	19.1	–7.2	1.1	0.9	0.7	–4.3	8.2	–3.2	0.1
AsH	2.9	2.1	–7.3	14.6	–5.2	0.8	0.5	0.4	–3.5	6.8	–2.9	0.1
SbH	2.1	1.6	–5.2	10.3	–3.5	0.5	0.0	–0.0	–1.6	3.0	–1.4	0.0
**Chalcogens**												
O	4.6	1.8	–22.0	40.0	–16.1	2.8	13.8	10.5	–36.1	76.8	–30.2	3.3
S	2.0	1.1	–9.2	17.2	–6.9	0.8	1.0	0.8	–6.4	11.8	–4.6	0.2
Se	1.3	0.8	–6.5	11.9	–4.5	0.5	0.2	0.1	–3.5	6.3	–2.6	0.1
Te	0.9	0.6	–4.5	8.1	–3.0	0.3	–0.2	–0.2	–1.2	2.0	–0.9	0.0

[a] Computed at ZORA-(U)OLYP/TZ2P.

[b] Stationary point does not exist.

When examining the variation of X down a group, the activation barriers for both addition@C and addition@X reactions can be explained by less destabilizing strain energies reinforced by more stabilizing interaction energies. The trend in interaction energy is largely set by the magnitude of Pauli repulsion. This trend is consistent across tetrels, pnictogens, and chalcogens, and it reflects a general stabilization as X becomes larger and more diffuse, leading to weaker orbital overlap and reduced steric repulsion.

When X is varied across a period, the activation barriers for the addition@C pathway are influenced primarily by the interaction energy, which itself is dominated by the orbital interaction and electrostatic potential terms. For elements in the second and third periods (e.g., CH_2 and NH), strain energy also contributes slightly to the observed trends. Notably, the orbital interaction term becomes increasingly stabilizing as the electronegativity of X increases, enhancing overlap between the radical SOMO and the substrate's orbitals. This effect is particularly strong for second-period elements such as O, where the π-HOMO and π -LUMO are highly polarized.

In the addition@X pathway, the activation barriers are likewise governed by the interaction energy term. However, unlike in the addition@C pathway, the interaction energy here is primarily determined by the Pauli repulsion term. As X becomes more electronegative (moving across the period), the increased polarization of the substrate's orbitals toward the X atom amplifies Pauli repulsion. This effect is most pronounced for O in the chalcogen group, where the addition@X barrier is significantly higher than for heavier chalcogens because of strong repulsive interactions.

Table S2. ASM and EDA energy terms (in kcal mol^{-1}) for the reaction energies of the addition@C and addition@X pathways.[a]

X	Addition@C						Addition@X					
	ΔE^{rxn}	ΔE^{rxn}_{int}	ΔE^{rxn}_{oi}	ΔE^{rxn}_{Pauli}	ΔV^{rxn}_{elstat}	ΔE^{rxn}_{strain}	ΔE^{rxn}	ΔE^{rxn}_{int}	ΔE^{rxn}_{oi}	ΔE^{rxn}_{Pauli}	ΔV^{rxn}_{elstat}	ΔE^{rxn}_{strain}
**Tetrels**												
CH_2	–22.2	–99.7	–270.0	316.2	–145.9	77.5	–22.2	–99.7	–270.0	316.2	–145.9	77.5
SiH_2	–49.5	–80.0	–245.3	297.3	–132.0	30.4	–48.5	–100.9	–193.2	196.9	–104.7	52.4
GeH_2	–54.7	–83.0	–251.8	303.6	–134.7	28.3	–41.3	–60.9	–145.8	179.0	–94.2	19.7
SnH_2	–65.3	–89.1	–257.9	307.6	–138.7	23.8	–41.9	–57.9	–125.2	151.0	–83.8	16.1
**Pnictogens**												
NH	–18.7	–80.1	–236.3	270.9	–114.7	61.4	–20.2	–43.9	–298.8	452.5	–197.6	23.7
PH	–42.8	–84.1	–250.0	296.3	–130.3	41.2	–30.7	–52.2	–184.8	255.6	–123.0	21.5
AsH	–51.1	–89.6	–259.0	302.4	–133.0	38.5	–26.2	–47.2	–147.9	202.4	–101.7	21.0
SbH	–59.6	–93.8	–264.9	309.9	–138.8	34.2	–27.8	–45.2	–123.4	164.9	–86.8	17.5
**Chalcogens**												
O	–12.9	–69.8	–220.5	247.7	–96.9	56.9	–8.3	–34.1	–278.9	427.2	–182.4	25.8
S	–39.5	–83.7	–243.0	274.3	–115.0	44.2	–30.8	–42.1	–214.3	323.5	–151.3	11.3
Se	–50.1	–90.6	–253.7	282.7	–119.5	40.5	–30.8	–41.1	–179.4	271.4	–133.1	10.3
Te	–59.7	–95.7	–261.4	292.1	–126.3	36.0	–32.9	–41.3	–154.1	231.5	–118.7	8.4

[a] Computed at ZORA-UOLYP/TZ2P.

Group-Specific Observations:

1. **Tetrels (CH_2, SiH_2, GeH_2, SnH_2):**

The Addition@C barriers decrease significantly down the group, driven by a marked reduction in strain energy and Pauli repulsion. This trend highlights the role of increasing orbital diffuseness in reducing steric effects and facilitating smoother interaction. For Addition@X, the barriers drop dramatically from SiH_2 to SnH_2, reflecting the same reduction in Pauli repulsion, although the magnitude of stabilization is smaller than for Addition@C.

2. **Pnictogens (NH, PH, AsH, SbH):**
For Addition@C, the barriers show a consistent decrease down the group, with the interaction energy remaining the dominant term. In the Addition@X pathway, the barriers are higher for NH due to strong Pauli repulsion but decrease substantially for heavier pnictogens. This indicates that larger, less electronegative X atoms mitigate repulsive interactions.
3. **Chalcogens (O, S, Se, Te):**
The Addition@C barriers decrease notably down the group as strain energy and Pauli repulsion are reduced. For O, the orbital interaction term strongly stabilizes the reaction, explaining its relatively high ΔE_{int} value despite the significant Pauli repulsion. For Addition@X, the barriers are highest for O due to strong Pauli repulsion and progressively decrease for S, Se, and Te, reflecting weaker repulsive forces and more diffuse orbitals.

These findings reinforce the importance of examining both group and period trends to fully understand the interplay of strain, interaction, and repulsion terms in determining activation barriers. The static analysis of transition states provides complementary insights that highlight the subtle yet critical role of electronic and geometric factors in shaping reaction energetics.

Table S3. Cartesian coordinates (Å), electronic energies (*E*), enthalpies (*H*), Gibb's free energies (*G*) (calculated at 298 K) and imaginary frequencies (N_{imag}, only for transition states) for all stationary points of the reaction of + $CH_3^{\bullet}$ + H_2C=X via both Addition@C and Addition@X calculated at ZORA-(U)OLYP/TZ2P.

R: $CH_3^{\bullet}$
E = –406.5 kcal mol^{-1}
H = –385.6 kcal mol^{-1}
G = –400.2 kcal mol^{-1}

C	-2.58567319	-0.51212304	-0.26136102
H	-1.51818211	-0.50525504	-0.44359403
H	-3.14328323	-1.43672810	-0.34481802
H	-3.09553222	0.40561403	0.00432500

R: H_2C=CH_2
E = –714.7 kcal mol^{-1}
H = –680.8 kcal mol^{-1}
G = –696.4 kcal mol^{-1}

C	-0.00000000	-0.00000000	0.66613605
C	0.00000000	-0.00000000	-0.66613605
H	0.00000000	0.92578307	-1.23773609
H	-0.00000000	0.92578307	1.23773609
H	-0.00000000	-0.92578307	1.23773609
H	0.00000000	-0.92578307	-1.23773609

TS: H_2C=CH_2 + $CH_3^{\bullet}$
E = –1113.6 kcal mol^{-1}
H = –1057.9 kcal mol^{-1}
G = –1078.7 kcal mol^{-1}
$\boldsymbol{v_{imag}}$ = 366*i* cm^{-1}

C	-1.43380510	0.20463501	0.54105104
C	-0.45925603	0.15914901	-0.40184903
C	1.67845312	-0.04472000	0.62152604
H	-1.89494714	-0.70103405	0.92734007
H	-1.74866913	1.14369908	0.98953007
H	1.53963411	-0.98043607	1.15123108
H	-0.09443001	1.07127708	-0.86544706
H	-0.24008902	-0.76594106	-0.92737607
H	2.28873616	-0.06338600	-0.27603702
H	1.68605312	0.86422706	1.21244109

P: H_2C=CH_2 + $CH_3^{\bullet}$
E = –1143.4 kcal mol^{-1}
H = –1085.4 kcal mol^{-1}
G = –1105.6 kcal mol^{-1}

C	-1.26956009	0.18498501	0.48428803
C	0.00793000	0.04140100	-0.26182902
C	1.26342809	0.01846600	0.61804804
H	-2.21548716	-0.08579101	0.02467600

H	-1.30191309	0.68301305	1.44927210
H	1.24371609	-0.82864606	1.31199809
H	0.10763501	0.86923406	-0.99125507
H	-0.02861200	-0.86541606	-0.88415306
H	2.16949416	-0.06500000	0.00864000
H	1.34504810	0.93522407	1.21272509

R: $H_2C{=}SiH_2$
E = –594.0 kcal mol^{-1}
H = –566.5 kcal mol^{-1}
G = –584.4 kcal mol^{-1}

C	-0.92654007	0.07067001	0.00000000
Si	0.77939906	-0.05941100	0.00000000
H	-1.57299911	-0.80298506	0.00000000
H	-1.43299810	1.03228107	0.00000000
H	1.48146511	-1.36610010	0.00000000
H	1.67167412	1.12554508	0.00000000

TS (Addition@C): $H_2C{=}SiH_2$ + $CH_3^{\bullet}$
E = –996.4 kcal mol^{-1}
H = –947.2 kcal mol^{-1}
G = –970.7 kcal mol^{-1}
ν_{imag} = 270*i* cm^{-1}

Si	-1.93545714	0.24148202	0.78597206
C	-0.64498005	0.17598501	-0.36651903
C	1.87009613	-0.06307300	0.69610005
H	-2.49175518	-0.98844807	1.40244310
H	-2.29022816	1.50073411	1.48643011
H	1.70397912	-1.00585807	1.20162409
H	-0.25279802	1.08034408	-0.82229006
H	-0.40055803	-0.74688605	-0.88416806
H	2.36138517	-0.06426700	-0.26944102
H	1.85078113	0.85212406	1.27404209

P (Addition@C): $H_2C{=}SiH_2$ + $CH_3^{\bullet}$
E = –1050.0 kcal mol^{-1}
H = –996.7 kcal mol^{-1}
G = –1017.8 kcal mol^{-1}

C	1.95211114	1.88597314	0.70535505
Si	1.13512408	1.48241211	-0.95817307
H	1.24528409	2.49585118	1.28276009
H	2.81360720	2.53541218	0.50286104
H	-0.12011901	0.68390505	-0.78658306
H	2.06173315	0.74169705	-1.87217913
C	2.39356017	0.66424405	1.53126111
H	3.12712623	0.05675400	0.99103107
H	1.54551511	0.01679600	1.77744913
H	2.85542221	0.97770307	2.47597118

TS (Addition@X): $H_2C{=}SiH_2 + CH_3^{\bullet}$
E = –1000.4 kcal mol^{-1}
H = –951.3 kcal mol^{-1}
G = –977.0 kcal mol^{-1}
$\boldsymbol{v_{imag}}$ = 58*i* cm^{-1}

C	-2.16233516	0.21952702	0.76858906
Si	-1.00584907	0.23492602	-0.49481004
C	2.36306617	-0.10595901	0.83645906
H	-2.60267919	-0.70288905	1.13803708
H	-2.47451518	1.12755908	1.27775209
H	2.04559715	-0.92982807	1.46294611
H	-0.42937603	1.49159011	-1.03043307
H	-0.60346604	-0.99444707	-1.21952109
H	2.79548320	-0.30258302	-0.13688601
H	2.35070217	0.90353607	1.22767709

P (Addition@X): $H_2C{=}SiH_2 + CH_3^{\bullet}$
E = –1048.9 kcal mol^{-1}
H = –997.8 kcal mol^{-1}
G = –1020.3 kcal mol^{-1}

C	0.63095105	-1.42163510	1.02551407
Si	-0.84861806	-0.82529406	0.08806801
C	-1.17707408	1.00835707	0.36789103
H	-0.31725102	1.61268712	0.05919500
H	-1.37846110	1.21778909	1.42358710
H	-2.04466115	1.33693910	-0.21410002
H	0.81936006	-2.47848318	1.20924109
H	1.40355210	-0.75494405	1.40613410
H	-2.04182615	-1.63689512	0.48636704
H	-0.64724105	-1.06952208	-1.37944310

R: $H_2C{=}GeH_2$
E = –561.2 kcal mol^{-1}
H = –534.3 kcal mol^{-1}
G = –552.8 kcal mol^{-1}

C	0.00000000	0.00000000	-0.97561207
Ge	0.00000000	0.00000000	0.80113106
H	0.00000000	0.92838807	-1.53703111
H	0.00000000	-0.92838807	-1.53703111
H	0.00000000	1.28304009	1.62427112
H	0.00000000	-1.28304009	1.62427112

TS (Addition@C): $H_2C{=}GeH_2 + CH_3^{\bullet}$
E = –964.3 kcal mol^{-1}
H = –915.7 kcal mol^{-1}
G = –940.5 kcal mol^{-1}
$\boldsymbol{v_{imag}}$ = 245*i* cm^{-1}

Ge	-2.02887115	0.25170702	0.79373106
C	-0.65497105	0.19047301	-0.36578703

C	1.93429114	-0.08511301	0.70389805
H	-2.59040219	-1.01863407	1.42816310
H	-2.36925717	1.53193811	1.55340811
H	1.74545613	-1.02346407	1.20919909
H	-0.27553102	1.10725608	-0.80465406
H	-0.43401303	-0.73002405	-0.89575906
H	2.40493417	-0.09437301	-0.27156602
H	1.91671114	0.83467606	1.27418509

P (Addition@C): $H_2C=GeH_2 + CH_3^{\bullet}$

E = –1022.4 kcal mol^{-1}

H = –969.8 kcal mol^{-1}

G = –991.9 kcal mol^{-1}

C	0.37574603	1.74128413	-1.27912309
Ge	-0.90954007	1.14408508	0.11875701
H	0.18461001	2.80558920	-1.45453810
H	1.37825610	1.66399612	-0.84426606
H	-2.37428317	1.28709709	-0.37283603
H	-0.66434505	-0.33913002	0.50322204
C	0.28668402	0.94831707	-2.58829319
H	1.01716507	1.32275310	-3.31805524
H	-0.70449505	1.02989907	-3.04657222
H	0.49441304	-0.11575601	-2.43428618

TS (Addition@X): $H_2C=GeH_2 + CH_3^{\bullet}$

E = –967.6 kcal mol^{-1}

H = –919.2 kcal mol^{-1}

G = –945.5 kcal mol^{-1}

$\boldsymbol{v_{imag}}$ = 56*i* cm^{-1}

C	-2.17970716	0.10519901	0.80451406
Ge	-0.99849507	0.33499102	-0.50712704
C	2.33794117	-0.11081801	0.85003906
H	-2.57048019	-0.87880006	1.04166407
H	-2.48504018	0.93321307	1.43570110
H	1.89877514	-0.38929103	1.79936313
H	-0.45857403	1.70771112	-0.89024206
H	-0.57553804	-0.79726106	-1.43537310
H	2.59768119	-0.87687806	0.12996901
H	2.62382819	0.91791707	0.66912205

P (Addition@X): $H_2C=GeH_2 + CH_3^{\bullet}$

E = –1008.9 kcal mol^{-1}

H = –958.4 kcal mol^{-1}

G = –982.0 kcal mol^{-1}

C	-2.23991816	0.99475607	1.00226807
Ge	-0.33248802	0.95377007	1.29894209
C	0.53294204	2.64375019	0.74966705
H	0.28085602	-0.20324601	0.48632204
H	-0.05881400	0.71541105	2.79621320

H	1.60999712	2.57880719	0.92907407
H	0.12650801	3.48007425	1.32413910
H	0.36035603	2.82736620	-0.31392602
H	-2.93757421	1.32583710	1.76835513
H	-2.68152919	0.71904505	0.04705400

R: $H_2C{=}SnH_2$
E = –524.1 kcal mol^{-1}
H = –498.7 kcal mol^{-1}
G = –519.2 kcal mol^{-1}

C	-2.00609614	0.51972804	1.20787709
Sn	-0.13475101	1.13392008	1.19474609
H	-2.24708116	-0.53790404	1.22551309
H	-2.82679720	1.22860409	1.23794509
H	1.20168109	0.07151801	1.14433008
H	0.31215302	2.78212620	1.16334808

TS (Addition@C): $H_2C{=}SnH_2$ + $CH_3^{\bullet}$
E = –928.7 kcal mol^{-1}
H = –881.9 kcal mol^{-1}
G = –908.6 kcal mol^{-1}
$\boldsymbol{v_{imag}}$ = 160*i* cm^{-1}

Sn	-2.41307217	0.28812802	0.88751406
C	-0.78865406	0.19517501	-0.27605402
C	2.07475115	-0.10419101	0.79751306
H	-2.99894722	-1.12066208	1.67414612
H	-2.74893720	1.70340412	1.79897413
H	1.87046313	-1.05158608	1.27881109
H	-0.41770603	1.10606108	-0.73945505
H	-0.57996104	-0.72299405	-0.81964506
H	2.47249018	-0.08730401	-0.20922902
H	2.03049715	0.81092706	1.37329410

P (Addition@C): $H_2C{=}SnH_2$ + $CH_3^{\bullet}$
E = –995.9 kcal mol^{-1}
H = –944.5 kcal mol^{-1}
G = –967.8 kcal mol^{-1}

C	-2.20248016	0.50693204	0.70881605
Sn	-0.09646601	1.12239608	0.93867107
H	-2.21713616	-0.25959002	-0.07271401
H	-2.73872920	1.37851910	0.31980502
H	0.83092606	-0.23235102	1.52927611
H	0.00231000	2.37127717	2.15304416
C	-2.83856620	-0.00568300	2.00286914
H	-2.31864717	-0.88577906	2.39655317
H	-3.88444628	-0.29775002	1.83220913
H	-2.84133820	0.75607005	2.79001520

P (Addition@X): $H_2C{=}SnH_2 + CH_3^{\bullet}$

E = –972.4 kcal mol^{-1}

H = –923.6 kcal mol^{-1}

G = –948.3 kcal mol^{-1}

C	-2.57523219	0.80149506	1.03759107
Sn	-0.48354503	0.40722603	1.01510707
H	-3.25956223	0.27965502	1.70446112
H	-3.03969322	1.52247311	0.36691903
H	0.01489700	-0.06674100	2.59834219
H	0.35272003	1.84201713	0.54423004
C	-0.00641400	-1.18454009	-0.38533303
H	1.07127208	-1.36736410	-0.37798003
H	-0.31542102	-0.89681506	-1.39310710
H	-0.52841704	-2.10032615	-0.09769601

R: $H_2C{=}NH$

E = –615.2 kcal mol^{-1}

H = –588.4 kcal mol^{-1}

G = –604.6 kcal mol^{-1}

C	-1.79411213	-0.11843201	0.73238505
N	-1.87604614	-1.12136808	-0.04464600
H	-2.72036620	0.34432202	1.09090308
H	-0.85679406	0.33766902	1.08575408
H	-0.93903007	-1.45268010	-0.30132802

TS (Addition@C): $H_2C{=}NH + CH_3^{\bullet}$

E = –1015.0 kcal mol^{-1}

H = –966.0 kcal mol^{-1}

G = –986.4 kcal mol^{-1}

$\boldsymbol{v_{imag}}$ = 356*i* cm^{-1}

N	-1.35329310	0.30377702	0.59491104
C	-0.48068303	0.14902501	-0.35342103
C	1.60666812	-0.05890400	0.62231304
H	-1.70256712	-0.61140704	0.90062506
H	1.57188311	0.82931306	1.24195609
H	1.49123211	-1.01776807	1.11527508
H	-0.11269501	1.04733608	-0.85573106
H	-0.29089502	-0.79884006	-0.87436706
H	2.21082716	-0.02682300	-0.27877302

P (Addition@C): $H_2C{=}NH + CH_3^{\bullet}$

E = –1040.4 kcal mol^{-1}

H = –989.4 kcal mol^{-1}

G = –1008.9 kcal mol^{-1}

C	-1.99448514	-0.04464100	0.68574705
N	-1.46439411	-0.79783906	-0.41829003
H	-2.21589516	0.97406707	0.33854402
H	-1.17492808	0.06773500	1.42641310
H	-1.48552411	-1.79001413	-0.13565601

C	-3.21304523	-0.64451805	1.40369510
H	-2.97578721	-1.61923212	1.84479513
H	-4.04619729	-0.77777506	0.70614905
H	-3.54820926	0.01957800	2.20810116

TS (Addition@X): H_2C=NH + $CH_3^{\bullet}$

E = –1013.0 kcal mol^{-1}

H = –964.1 kcal mol^{-1}

G = –984.7 kcal mol^{-1}

$\boldsymbol{v_{imag}}$ = 347*i* cm^{-1}

C	-3.33611224	0.27433802	1.47505111
N	-2.38741217	0.60382404	0.65812505
H	-4.30976531	-0.12210001	1.16428808
H	-3.15543823	0.35726003	2.54906218
C	-0.55562204	-0.61613904	0.52681804
H	-0.03705300	-0.07225901	-0.25502402
H	-0.20775901	-0.46993503	1.54226511
H	-0.96952907	-1.58713411	0.27483102
H	-2.68218319	0.47714703	-0.31284602

P (Addition@X): H_2C=NH + $CH_3^{\bullet}$

E = –1041.9 kcal mol^{-1}

H = –990.2 kcal mol^{-1}

G = –1009.6 kcal mol^{-1}

C	-0.36044403	-0.72964905	2.13279815
N	-0.22439002	0.15663601	3.18888923
C	-1.23854209	0.21050102	4.22499830
H	-1.36336910	-0.89227406	1.75179813
H	-1.00379507	1.01695907	4.92556835
H	0.48110003	-0.81356006	1.45420410
H	0.71974905	0.23762402	3.54270026
H	-1.33486210	-0.73303005	4.78903734
H	-2.21102916	0.43136803	3.77109427

R: H_2C=PH

E = –529.8 kcal mol^{-1}

H = –506.7 kcal mol^{-1}

G = –524.0 kcal mol^{-1}

C	1.32010010	0.19803001	-1.70728212
P	0.39215003	0.12658101	-0.31941302
H	-0.89094606	0.08003301	-0.95881707
H	2.40446717	0.24652502	-1.61525812
H	0.93421007	0.20086901	-2.72409820

TS (Addition@C): H_2C=PH + $CH_3^{\bullet}$

E = –932.6 kcal mol^{-1}

H = –887.6 kcal mol^{-1}

G = –910.0 kcal mol^{-1}

$\boldsymbol{v_{imag}}$ = 249*i* cm^{-1}

P	-1.82975013	0.39216503	0.84896206
C	-0.65215105	0.15150601	-0.34238602
C	1.78185713	-0.05946800	0.68054505
H	-2.12232015	-0.99028307	1.09189008
H	1.76142113	0.87084506	1.23377509
H	1.60915712	-0.98644307	1.21275209
H	-0.23806102	1.02172807	-0.84639106
H	-0.41116603	-0.80800006	-0.79018406
H	2.29297517	-0.09104801	-0.27454502

P (Addition@C): $H_2C{=}PH + CH_3^{\bullet}$

E = –979.1 kcal mol^{-1}

H = –930.6 kcal mol^{-1}

G = –951.2 kcal mol^{-1}

C	1.31483209	0.35320803	-1.70536412
P	0.45639803	-0.46796303	-0.26892002
H	-0.75343605	0.29253402	-0.37833803
H	2.30116117	-0.11922201	-1.78587313
H	0.76622806	0.08664501	-2.61942019
C	1.46242411	1.87721714	-1.60771812
H	2.00508014	2.16948216	-0.70282405
H	0.48572803	2.37099617	-1.58585911
H	2.01727815	2.26628416	-2.46997218

TS (Addition@X): $H_2C{=}PH + CH_3^{\bullet}$

E = –935.4 kcal mol^{-1}

H = –891.3 kcal mol^{-1}

G = –913.5 kcal mol^{-1}

$\boldsymbol{v_{imag}}$ = 100*i* cm^{-1}

C	-1.80125413	0.35252603	0.34944903
P	-0.53832304	-0.06196400	-0.66988505
C	2.30994717	-0.14477101	0.73314505
H	-2.34901617	-0.43851803	0.85976206
H	-2.12903915	1.36679010	0.56509904
H	2.19045916	-1.17118808	1.05481208
H	-0.17521801	1.26568709	-1.06888308
H	2.08164015	0.66242405	1.41756910
H	2.82190820	0.06823600	-0.19701901

P (Addition@X): $H_2C{=}PH + CH_3^{\bullet}$

E = –967.0 kcal mol^{-1}

H = –920.2 kcal mol^{-1}

G = –941.1 kcal mol^{-1}

P	-1.38266710	-0.03325500	0.47794403
C	-0.92596607	0.24125002	-1.19492109
C	0.19118801	0.06470800	1.45647710
H	-1.59996212	-1.44207910	0.46758403
H	0.55041204	1.09776408	1.43268910
H	-0.02783500	-0.19185701	2.49713518

H	-0.34154402	1.12077708	-1.45434110
H	-1.52952911	-0.19091301	-1.98878114
H	0.96939907	-0.59652204	1.06751708

R: H_2C=AsH
E = –503.5 kcal mol^{-1}
H = –481.2 kcal mol^{-1}
G = –499.4 kcal mol^{-1}

C	0.31904475	0.65967485	1.70195533
As	1.75145080	-0.34446909	1.32740779
H	-0.71099325	0.33509431	1.58564997
H	0.46666440	1.67142192	2.07447533
H	0.95771350	-1.57905194	0.87271212

TS (Addition@C): H_2C=AsH + $CH_3^{\bullet}$
E = –907.1 kcal mol^{-1}
H = –863.0 kcal mol^{-1}
G = –886.7 kcal mol^{-1}
ν_{imag} = 214*i* cm^{-1}

As	-1.96959614	0.39575003	0.92308007
C	-0.70485305	0.16485701	-0.34889903
C	1.84248213	-0.05881100	0.69873905
H	-2.24624416	-1.10140508	1.12854108
H	1.84664313	0.90816507	1.18525309
H	1.63339912	-0.94293207	1.28713809
H	-0.28538802	1.04265908	-0.83300806
H	-0.45176703	-0.79336906	-0.78978006
H	2.32156017	-0.16712901	-0.26684702

P (Addition@C): H_2C=AsH + $CH_3^{\bullet}$
E = –961.1 kcal mol^{-1}
H = –913.2 kcal mol^{-1}
G = –934.8 kcal mol^{-1}

C	0.39357703	0.68110705	1.79342113
As	2.06857815	-0.38052403	1.53654711
H	-0.38464303	-0.02172400	2.11345615
H	0.59526904	1.34785310	2.63895019
H	1.49122011	-1.20991409	0.37836403
C	-0.06449300	1.47695811	0.57037904
H	-0.96170107	2.06406115	0.80491906
H	0.70688705	2.17609916	0.23063202
H	-0.30833402	0.81762406	-0.26870902

TS (Addition@X): H_2C=AsH + $CH_3^{\bullet}$
E = –909.4 kcal mol^{-1}
H = –865.6 kcal mol^{-1}
G = –890.8 kcal mol^{-1}
ν_{imag} = 77*i* cm^{-1}

C	-1.87241313	0.45079403	0.34683002
As	-0.57366904	-0.19219201	-0.71037105
C	2.41134517	-0.17963901	0.76259505
H	-2.43771718	-0.24135102	0.96752507
H	-2.14019515	1.50036411	0.42763003
H	2.48792318	-1.25927509	0.74371705
H	-0.16789001	1.16693608	-1.29978109
H	2.03533915	0.31725902	1.64769412
H	2.85663021	0.40452103	-0.03308900

P (Addition@X): $H_2C{=}AsH + CH_3^{\bullet}$

E = –936.2 kcal mol^{-1}

H = –890.4 kcal mol^{-1}

G = –912.6 kcal mol^{-1}

C	1.03393607	-0.13284101	-0.27607402
As	-0.80925606	-0.57144004	-0.49292704
H	-1.35105810	0.84956706	-0.30938202
C	-1.24305309	-1.25102009	1.33160910
H	1.72606412	-0.89364806	0.07690101
H	-0.91566007	-0.55631804	2.10572715
H	1.45797310	0.70710305	-0.81979906
H	-0.74382505	-2.21432516	1.46097711
H	-2.32360317	-1.40268710	1.39185610

R: $H_2C{=}SbH$

E = –474.4 kcal mol^{-1}

H = –453.1 kcal mol^{-1}

G = –472.0 kcal mol^{-1}

C	0.54789304	-1.40462110	0.54413904
Sb	-0.27168302	0.37307703	0.20034801
H	-1.84920713	-0.10028401	0.72795305
H	0.00266100	-2.25946916	0.93583307
H	1.60403012	-1.56877811	0.33904902

TS (Addition@C): $H_2C{=}SbH + CH_3^{\bullet}$

E = –878.8 kcal mol^{-1}

H = –835.8 kcal mol^{-1}

G = –860.7 kcal mol^{-1}

$\boldsymbol{v_{imag}}$ = 174*i* cm^{-1}

Sb	-2.20913716	0.43593503	1.04384708
C	-0.79076106	0.13314101	-0.34116902
C	1.93766814	-0.04406900	0.71019505
H	-2.47615618	-1.24957309	1.32577610
H	1.90931914	0.90603707	1.22766009
H	1.75200813	-0.95624107	1.26224609
H	-0.35700003	0.98008607	-0.86667406
H	-0.51644404	-0.84797906	-0.71654405
H	2.37028617	-0.09890101	-0.28102802

P (Addition@C): H_2C=SbH + $CH_3^•$

E = –940.5 kcal mol$^{–1}$

H = –893.3 kcal mol$^{–1}$

G = –915.6 kcal mol$^{–1}$

C	0.79901706	-1.36996410	1.02607507
Sb	-0.27096202	-0.12567801	-0.44587603
H	-1.79970713	-0.83946906	-0.06442800
H	0.39819203	-1.09470208	2.00782514
H	1.83909213	-1.02785607	0.99300807
C	0.70919305	-2.87921021	0.79990406
H	1.10323008	-3.16982123	-0.18001101
H	-0.32408502	-3.23606023	0.85901206
H	1.28919209	-3.42178525	1.55855511

TS (Addition@X): H_2C=SbH + $CH_3^•$

E = –880.9 kcal mol$^{–1}$

H = –838.6 kcal mol$^{–1}$

G = –863.6 kcal mol$^{–1}$

$\boldsymbol{v_{imag}}$ = 43*i* cm$^{–1}$

C	-2.16908716	-0.95715007	-0.49517004
Sb	-0.71784905	0.37410603	-0.78036706
C	2.59479119	-0.97808807	0.59847304
H	-2.36974917	-1.79050013	-1.16355008
H	-2.81416920	-0.88284506	0.37816503
H	2.21715216	-1.98965814	0.51970004
H	2.49743218	-0.43644203	1.53088411
H	-0.16502801	-0.33909002	-2.25549316
H	3.15478023	-0.54533404	-0.22121702

P (Addition@X): H_2C=SbH + $CH_3^•$

E = –908.7 kcal mol$^{–1}$

H = –864.1 kcal mol$^{–1}$

G = –887.5 kcal mol$^{–1}$

C	0.61636704	-1.62644612	0.83293806
Sb	-0.23906302	-0.45393503	-0.70073605
C	-1.27693009	0.99065707	0.57970404
H	0.66257205	-2.71032220	0.75093105
H	1.27552209	-1.17187408	1.57056911
H	-1.60208812	-1.49077611	-0.92481507
H	-0.52993604	1.64285912	1.03824407
H	-1.84685113	0.47412603	1.35272010
H	-1.94345114	1.59195911	-0.04329500

R: H_2C=O

E = –500.0 kcal mol$^{–1}$

H = –481.4 kcal mol$^{–1}$

G = –497.3 kcal mol$^{–1}$

C	-2.21587316	-0.54245704	-0.33083602
O	-1.04497808	-0.29294102	-0.48380403

H	-2.62350819	-1.57192811	-0.46041403
H	-2.95671421	0.24222102	-0.05122800

TS (Addition@C): $H_2C{=}O + CH_3^{\bullet}$
E = –901.8 kcal mol^{-1}
H = –860.9 kcal mol^{-1}
G = –881.0 kcal mol^{-1}
$\boldsymbol{v_{imag}}$ = 297*i* cm^{-1}

C	-0.59409720	0.22198757	0.00823027
C	-2.59411471	1.26828466	-0.05676673
O	0.23044054	1.09231607	-0.27274159
H	-0.96721324	-0.49251875	-0.75745277
H	-0.80180693	-0.06184352	1.06282940
H	-2.40139358	2.01388254	0.70588967
H	-3.23248543	0.42852032	0.19851503
H	-2.55903739	1.58714456	-1.09201630

P (Addition@C): $H_2C{=}O + CH_3^{\bullet}$
E = –919.4 kcal mol^{-1}
H = –876.7 kcal mol^{-1}
G = –896.0 kcal mol^{-1}

C	-0.97466007	0.41148503	-0.00230100
C	-2.32077417	1.13249908	-0.04974000
O	0.13331801	1.13654708	-0.27332202
H	-0.96652107	-0.48002503	-0.67738805
H	-0.81535406	-0.08793601	0.98557907
H	-2.34084417	1.95422014	0.67295705
H	-3.13803223	0.44158703	0.18700901
H	-2.49684018	1.54939611	-1.04630708

TS (Addition@X): $H_2C{=}O + CH_3^{\bullet}$
E = –892.6 kcal mol^{-1}
H = –851.8 kcal mol^{-1}
G = –872.3 kcal mol^{-1}
$\boldsymbol{v_{imag}}$ = 467*i* cm^{-1}

C	-0.38754401	-0.34566817	-0.09497618
C	-0.53819254	2.43695398	-0.51733630
O	0.22223959	0.62503628	-0.60705184
H	-0.91612451	-1.07913828	-0.72786808
H	-0.47501758	-0.46086012	0.99977643
H	-0.69202326	2.54839243	0.55061517
H	-1.41950297	2.31199148	-1.13668271
H	0.29567663	2.97541746	-0.95198192

P (Addition@X): $H_2C{=}O + CH_3^{\bullet}$
E = –914.8 kcal mol^{-1}
H = –871.1 kcal mol^{-1}
G = –890.5 kcal mol^{-1}

C	-3.16644923	-0.08028301	1.41911010
O	-2.26358916	0.17114701	0.43634803
H	-4.12622330	0.39653703	1.26001109
H	-2.80967320	-0.34215302	2.41285917
C	-0.96457207	-0.37707203	0.64715605
H	-0.35411003	-0.07605501	-0.20677101
H	-0.51862504	0.01514600	1.57196611
H	-1.00729107	-1.47318711	0.70146205

R: $H_2C{=}S$
E = –425.3 kcal mol^{-1}
H = –407.7 kcal mol^{-1}
G = –424.6 kcal mol^{-1}

S	-0.52992704	1.96507114	-0.49979904
C	-1.09954208	3.38966924	-0.99215207
H	-2.17245616	3.59625426	-1.06354908
H	-0.43947203	4.21540630	-1.27753409

TS (Addition@C): $H_2C{=}S$ + $CH_3^{\bullet}$
E = –829.8 kcal mol^{-1}
H = –790.3 kcal mol^{-1}
G = –812.2 kcal mol^{-1}
$\boldsymbol{v_{imag}}$ = 188*i* cm^{-1}

S	-1.74527913	0.23428602	0.73312905
C	-0.59698904	0.16999001	-0.42121303
C	1.80297313	-0.05652200	0.67603905
H	2.35596817	-0.06090000	-0.25592302
H	1.72777412	0.86292906	1.24252809
H	1.58777911	-0.99648507	1.16815908
H	-0.17024701	1.07444008	-0.86170406
H	-0.32221302	-0.76773706	-0.91051507

P (Addition@C): $H_2C{=}S$ + $CH_3^{\bullet}$
E = –871.3 kcal mol^{-1}
H = –828.3 kcal mol^{-1}
G = –848.2 kcal mol^{-1}

S	-1.32774510	1.90948214	-1.36967110
C	-1.03698207	3.60389426	-0.83851306
H	-1.69849812	3.76583927	0.02619000
H	-0.02154600	3.61668526	-0.41399403
C	-1.21591609	4.69564234	-1.88958814
H	-1.01403307	5.68081341	-1.45083010
H	-2.23640716	4.69987934	-2.28336316
H	-0.53111204	4.54941133	-2.72992620

TS (Addition@X): $H_2C{=}S$ + $CH_3^{\bullet}$
E = –830.8 kcal mol^{-1}
H = –792.2 kcal mol^{-1}

G = –814.3 kcal mol^{-1}
$\boldsymbol{v_{imag}}$ = 116*i* cm^{-1}

C	-1.94010014	0.23240002	0.81288606
S	-0.79248906	0.10296101	-0.32187202
C	1.98016614	-0.06222200	0.67769205
H	-2.49028418	-0.63719605	1.18464209
H	-2.20382716	1.19156809	1.26858509
H	1.82175313	-1.01717807	1.16192608
H	2.37203217	-0.03306000	-0.33118202
H	1.93235414	0.85044306	1.25743509

P (Addition@X): $H_2C{=}S + CH_3^{\bullet}$
E = –862.6 kcal mol^{-1}
H = –821.0 kcal mol^{-1}
G = –841.8 kcal mol^{-1}

S	-1.82635513	1.84686213	-1.28442209
C	-1.29047209	3.40589525	-0.84936306
H	-1.40931210	3.68078026	0.19188401
H	-1.07629708	4.16142930	-1.59641611
C	-1.34962710	1.73545612	-3.02799422
H	-0.26426002	1.81310913	-3.13446523
H	-1.84286413	2.51733618	-3.61226726
H	-1.68237612	0.75714305	-3.38243724

R: $H_2C{=}Se$
E = –402.3 kcal mol^{-1}
H = –385.1 kcal mol^{-1}
G = –402.8 kcal mol^{-1}

C	0.15065701	-0.01691000	0.00000000
Se	-1.59317411	0.17844201	0.00000000
H	0.82441606	0.84114706	0.00000000
H	0.61810204	-1.00267907	0.00000000

TS (Addition@C): $H_2C{=}Se + CH_3^{\bullet}$
E = –807.5 kcal mol^{-1}
H = –768.4 kcal mol^{-1}
G = –791.8 kcal mol^{-1}
$\boldsymbol{v_{imag}}$ = 148*i* cm^{-1}

Se	-1.92501214	0.28721102	0.78523706
C	-0.66594505	0.16306101	-0.45250803
C	1.88391714	-0.05551400	0.70445905
H	2.42488917	-0.00563400	-0.23278702
H	1.76128213	0.84106706	1.29818809
H	1.67598212	-1.01982707	1.15008608
H	-0.22053502	1.04939508	-0.90245706
H	-0.38818803	-0.79540206	-0.88937806

P (Addition@C): $H_2C{=}Se + CH_3^{\bullet}$
E = –858.9 kcal mol^{-1}

H = –815.9 kcal mol^{-1}
G = –836.6 kcal mol^{-1}

C	0.18197201	-1.21848709	0.89586406
Se	-1.32416410	-0.40849103	1.85506813
H	-0.07305101	-1.12023708	-0.16652901
H	1.02668007	-0.54019904	1.06824808
C	0.51836804	-2.65490219	1.27086309
H	1.37022210	-3.00970022	0.67587605
H	-0.32645202	-3.32413124	1.08423608
H	0.78611306	-2.73897220	2.32806717

TS (Addition@X): H_2C=Se + $CH_3^{\bullet}$
E = –808.6 kcal mol^{-1}
H = –770.4 kcal mol^{-1}
G = –793.8 kcal mol^{-1}
v_{imag} = 65i cm^{-1}

C	2.43101882	-0.52472369	-0.05373944
Se	0.97799714	-1.49918608	-0.24675918
C	-1.79552964	0.25466658	0.05296491
H	2.95322019	-0.09880237	-0.91108267
H	2.84710932	-0.30673860	0.93043334
H	-2.44983119	-0.60720872	0.00904653
H	-1.57347241	0.80799305	-0.85041215
H	-1.48616909	0.64471163	1.01399733

P (Addition@X): H_2C=Se + $CH_3^{\bullet}$
E = –839.6 kcal mol^{-1}
H = –798.6 kcal mol^{-1}
G = –820.6 kcal mol^{-1}

C	1.43073210	0.94311607	1.31305609
Se	0.33975302	0.00390400	0.14695901
C	0.75606005	0.95704807	-1.51556811
H	1.43007010	0.61430204	2.34543117
H	1.81445413	1.91955514	1.04305208
H	0.16782501	0.48332903	-2.30365417
H	1.82055313	0.86220406	-1.73469112
H	0.47142703	2.00609214	-1.42014510

R: H_2C=Te
E = –378.6 kcal mol^{-1}
H = –361.8 kcal mol^{-1}
G = –380.1 kcal mol^{-1}

C	-1.19990509	1.02532407	2.43903118
Te	-0.38110403	0.42063303	0.76340005
H	-2.28048116	1.07053808	2.56432518
H	-0.59780904	1.33325310	3.29231424

TS (Addition@C): H_2C=Te + $CH_3^{\bullet}$
E = –784.2 kcal mol^{-1}

H = –745.6 kcal mol⁻¹
G = –770.0 kcal mol⁻¹
$\boldsymbol{v_{imag}}$ = 123*i* cm⁻¹

C	-0.87736506	1.28786109	1.96388014
Te	-0.32780202	0.66635505	0.17229701
H	-1.85336313	1.73526712	2.13601015
H	-0.18411701	1.29450009	2.80164220
C	-2.11926515	-0.82525606	3.66290026
H	-1.21258909	-1.41568510	3.69251227
H	-2.88007621	-1.05008508	2.92671121
H	-2.34238117	-0.14332801	4.47421032

P (Addition@C): $H_2C{=}Te + CH_3^{\bullet}$
E = –844.8 kcal mol⁻¹
H = –801.9 kcal mol⁻¹
G = –823.1 kcal mol⁻¹

C	-1.28871917	0.83639291	2.54020977
Te	-0.52122182	0.69219079	0.50810646
H	-2.08939380	1.58069353	2.47565979
H	-0.47358410	1.29885502	3.10669068
C	-1.76234781	-0.46494502	3.17163050
H	-0.95442313	-1.19943281	3.23728459
H	-2.57819604	-0.91739370	2.60050872
H	-2.12907297	-0.27673161	4.19007208

TS (Addition@X): $H_2C{=}Te + CH_3^{\bullet}$
E = –785.3 kcal mol⁻¹
H = –746.9 kcal mol⁻¹
G = –773.8 kcal mol⁻¹
$\boldsymbol{v_{imag}}$ = 17*i* cm⁻¹

C	-1.55866711	1.43487010	2.73549220
Te	-0.69005705	1.30528909	0.98073107
H	-2.63171519	1.59081311	2.83289220
H	-0.99394707	1.34429010	3.66183926
C	-0.46249503	-2.28713016	-0.58767304
H	0.30125902	-1.96198214	-1.28315109
H	-1.50667011	-2.23938216	-0.86977606
H	-0.17743901	-2.71472920	0.36531503

P (Addition@X): $H_2C{=}Te + CH_3^{\bullet}$
E = –818.0 kcal mol⁻¹
H = –777.6 kcal mol⁻¹
G = –800.6 kcal mol⁻¹

C	-1.16363808	0.72201305	2.28061416
Te	0.16977801	0.37189303	0.77505206
H	-2.14153015	0.25377702	2.26724716
H	-0.97603007	1.56252311	2.93927021
C	-0.79267906	-1.39628310	-0.00624400
H	-0.19697401	-1.74110113	-0.85316906

H	-1.79933013	-1.14027208	-0.33796102
H	-0.81932706	-2.16051116	0.77086006
